# Simple and Rapid Discrimination of Methicillin-Resistant Staphylococcus aureus Based on Gram Staining and Machine Vision

**DOI:** 10.1128/spectrum.05282-22

**Published:** 2023-07-03

**Authors:** Menghuan Yu, Haimei Shi, Hao Shen, Xueqin Chen, Li Zhang, Jianhua Zhu, Guoqing Qian, Bin Feng, Shaoning Yu

**Affiliations:** a Institute of Mass Spectrometry, School of Material Science and Chemical Engineering, Ningbo University, Ningbo, Zhejiang, China; b Department of Intensive Care Unit, The First Affiliated Hospital of Ningbo University, Ningbo, Zhejiang, China; c Department of Clinical Lab, Peking Union Medical College Hospital, Peking Union Medical College & Chinese Academy Medical Science, Beijing, China; University of Guelph College of Biological Science

**Keywords:** MRSA, MSSA, Gram stain, machine vision

## Abstract

Methicillin-resistant Staphylococcus aureus (MRSA) is a clinical threat with high morbidity and mortality. Here, we describe a new simple, rapid identification method for MRSA using oxacillin sodium salt, a cell wall synthesis inhibitor, combined with Gram staining and machine vision (MV) analysis. Gram staining classifies bacteria as positive (purple) or negative (pink) according to the cell wall structure and chemical composition. In the presence of oxacillin, the integrity of the cell wall for methicillin-susceptible S. aureus (MSSA) was destroyed immediately and appeared Gram negative. In contrast, MRSA was relatively stable and appeared Gram positive. This color change can be detected by MV. The feasibility of this method was demonstrated in 150 images of the staining results for 50 clinical S. aureus strains. Based on effective feature extraction and machine learning, the accuracies of the linear linear discriminant analysis (LDA) model and nonlinear artificial neural network (ANN) model for MRSA identification were 96.7% and 97.3%, respectively. Combined with MV analysis, this simple strategy improved the detection efficiency and significantly shortened the time needed to detect antibiotic resistance. The whole process can be completed within 1 h. Unlike the traditional antibiotic susceptibility test, overnight incubation is avoided. This new strategy could be used for other bacteria and represents a new rapid method for detection of clinical antibiotic resistance.

**IMPORTANCE** Oxacillin sodium salt destroys the integrity of the cell wall of MSSA immediately, appearing Gram negative, whereas MRSA is relatively stable and still appears Gram positive. This color change can be detected by microscopic examination and MV analysis. This new strategy has significantly reduced the time to detect resistance. The results show that using oxacillin sodium salt combined with Gram staining and MV analysis is a new, simple and rapid method for identification of MRSA.

## INTRODUCTION

Staphylococcus aureus is a major cause of hospital- and community-acquired infections, resulting in endocarditis, bacteremia, osteomyelitis, and skin and soft tissue infections (1). Antibiotics are effective means of clinically inhibiting pathogenic bacteria (2). A primary and clinical issue associated with S. aureus is the remarkable ability to acquire resistance to multiple antibiotics (3). The emergence of resistance in S. aureus was due to the introduction of penicillin within 2 years. The first penicillin-resistant S. aureus strain was detected in 1942 (4). Methicillin-resistant S. aureus (MRSA) was first reported in 1961 (3), shortly after the semisynthetic antibiotic methicillin was introduced (3). MRSA is a terrible clinical threat with high morbidity and mortality, much higher than those of methicillin-susceptible S. aureus (MSSA) (4). The current “gold standard” for clinical detection of MRSA is the phenotypic method of antibiotic sensitivity tests (ASTs), such as the disk diffusion test and broth dilution test (5). ASTs can infer the concentration of antibiotics required to inhibit microbial multiplication *in vitro* (6). Although these methods can accurately guide antibiotic use, they require bacterial culture and generally take 2 to 3 days (7–9). Genotypic methods (usually DNA based, amplification based, or sequencing based) can rapidly detect specific antibiotic resistance genes (6). However, nucleic acid amplification technology (NAAT) can only find resistance if it is looking for it, and genes that are potentially found may not be derived from the actual causative organism (10). In addition, NAAT neither defines the MIC nor directly indicates which antibiotic should be used (10). Quantitative reverse transcription-PCR (qRT-PCR) can provide rough MIC values, but the costs of equipment and reagents for qRT-PCR far exceeds those of conventional ASTs (11). In addition, there are other FDA-approved technologies, such as matrix-assisted laser desorption ionization–time of flight mass spectrometry (MALDI-TOF MS) (12), multiplexed automated digital microscopy (MADM) (5), and the fluorescence *in situ* hybridization-based Accelerate Pheno system for AST (13). Although most of these methods are highly sensitive, they have complicated sample preprocessing steps and expensive and complex instruments. Fourier transform infrared (FTIR) spectroscopy has also been used to detect bacterial susceptibility to antibiotics, but the FTIR signals of bacteria are disturbed by many environmental factors, especially water (14, 15). In addition, biosensor-based technologies for detecting changes in microbial metabolism or movement have not yet provided convincing clinical evidence (16). Thus, rapid and low-cost identification methods are still urgently needed for clinical identification of MRSA.

Gram stain is a significant microbial classification tool. It separates bacteria into Gram-positive (purple) and Gram-negative (pink) bacteria based on the chemical and structural composition of their cell walls (17, 18). The addition of antibiotics that inhibit cell wall synthesis, such as penicillin, oxacillin, and vancomycin, to growing cultures of bacteria can effectively inhibit the synthesis of cell wall peptidoglycan (19, 20). In the presence of antibiotics, the integrity of the cell wall of Gram-positive bacteria is damaged. The complex of crystal violet-iodine is not retained during the decolorization process and appears Gram negative (17).

Artificial intelligence (AI) is increasingly being applied in health care, including in clinical diagnosis and prognosis (21). Many researchers think that AI can work as well or more accurately than clinicians in medical practice (22). Goh et al. pointed out that AI algorithms are more predictive than clinicians in early diagnosis (23). Many researchers use AI for bacterial detection. Lee et al. developed a method for rapid detection of bacteria in urine using laser scattering combined with deep learning analysis (24). Wang et al. indicated that a combination of Raman spectroscopy and deep learning algorithms can accurately identify bacteria at the genus and species levels (25). Machine vision (MV) technology belongs to a subfield of AI that has made great leaps in recent years (26). In MV, visual information is captured by a camera, converted to a digital format, and processed by a machine learning algorithm, such as an artificial neural network (ANN) (27). MV technology plays an important role in biomedical image analysis. Esteva et al. concluded that the development of MV technology has mainly been in medical imaging (28), such as cardiology (29), pathology (30), and dermatology (31). Due to the large amount of image data, manual processing is time-consuming, inefficient, and error-prone. Computerized approaches have a unified standard, which can significantly shorten time, increase the efficiency of image data processing, and avoid the risk of human errors in judgment (32, 33).

**FIG 1:**
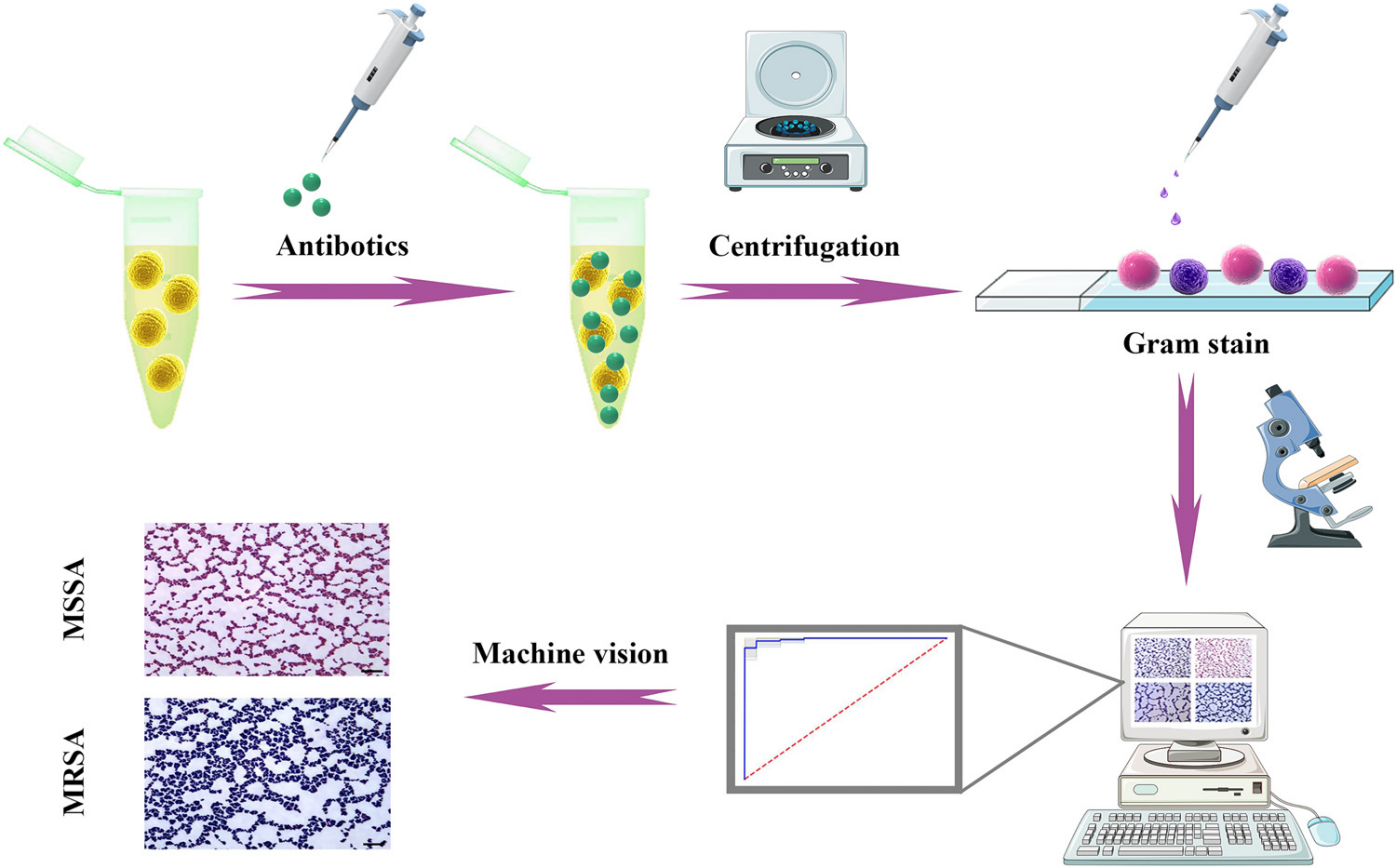
Schematic of the identification of MRSA by oxacillin sodium salt treatment combined with Gram staining and MV analysis.

We describe a new method for rapidly distinguishing MSSA and MRSA using oxacillin sodium salt, followed by Gram staining and MV analysis (Fig. 1). After short-term treatment with oxacillin sodium salt, the staining results are different for MRSA and MSSA, which can be easily distinguished by MV analysis.

## RESULTS

### Optimization of oxacillin sodium salt concentration.

**FIG 2:**
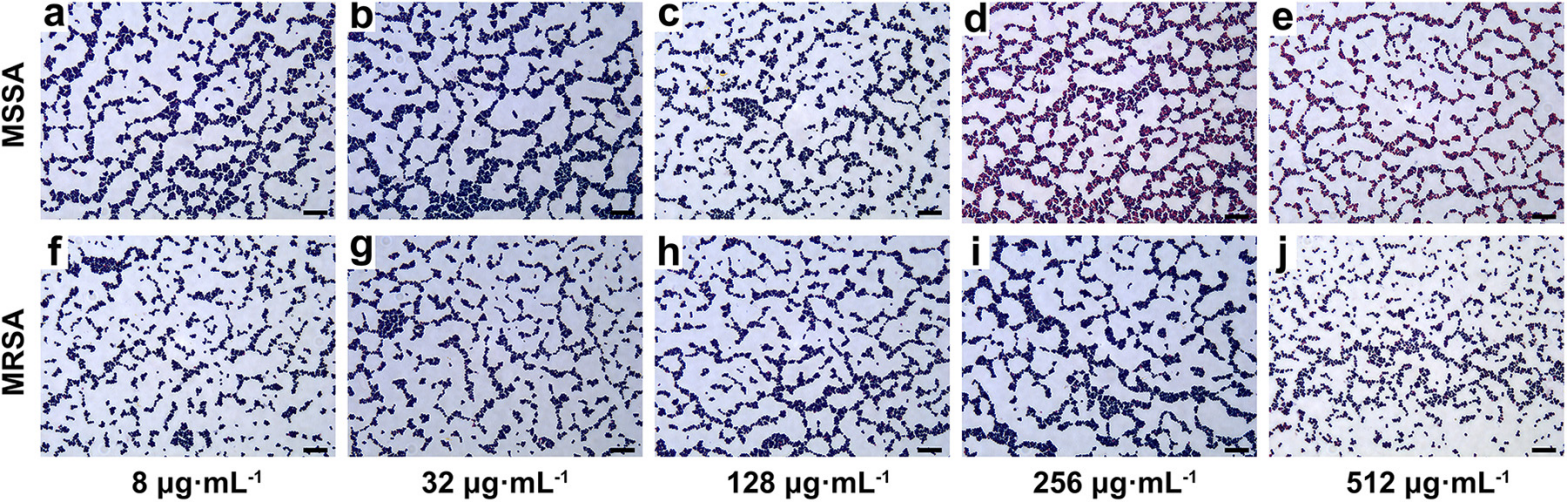
Microscopic images of MSSA strain 21B09710 (a to e) and MRSA strain 21B07569 (f to j) at 8, 32, 128, 256, and 512 μg mL^−1^ oxacillin sodium salt. Scale bars = 10 µm.

The microscope images of the staining of MSSA strain 21B09710 and MRSA strain 21B07569 incubated with 8, 32, 128, 256, and 512 μg mL^−1^ oxacillin sodium salt for 30 min are shown in Fig. 2. When the concentration of oxacillin sodium salt was 8, 32, or 128 μg mL^−1^, the purple cocci under the microscope field accounted for most of the MSSA and MRSA cells (Fig. 2a to c and Fig. 2f to h); thus, MSSA and MRSA could not be distinguished. However, the proportions of pink spheres in Fig. 2d and e were higher than those in Fig. 2i and j when the concentrations of oxacillin sodium salt were 256 and 512 μg mL^−1^, respectively. So, MSSA and MRSA could be clearly identified at higher concentrations of oxacillin sodium salt. However, the proportion of pink spheres in MRSA increased in Fig. 2j, which may be due to the high concentration of antibiotics. To avoid observation errors and save drugs, 256 μg mL^−1^ oxacillin sodium salt was finally selected.

### Optimization of incubation time for oxacillin sodium salt and bacteria.

**FIG 3:**
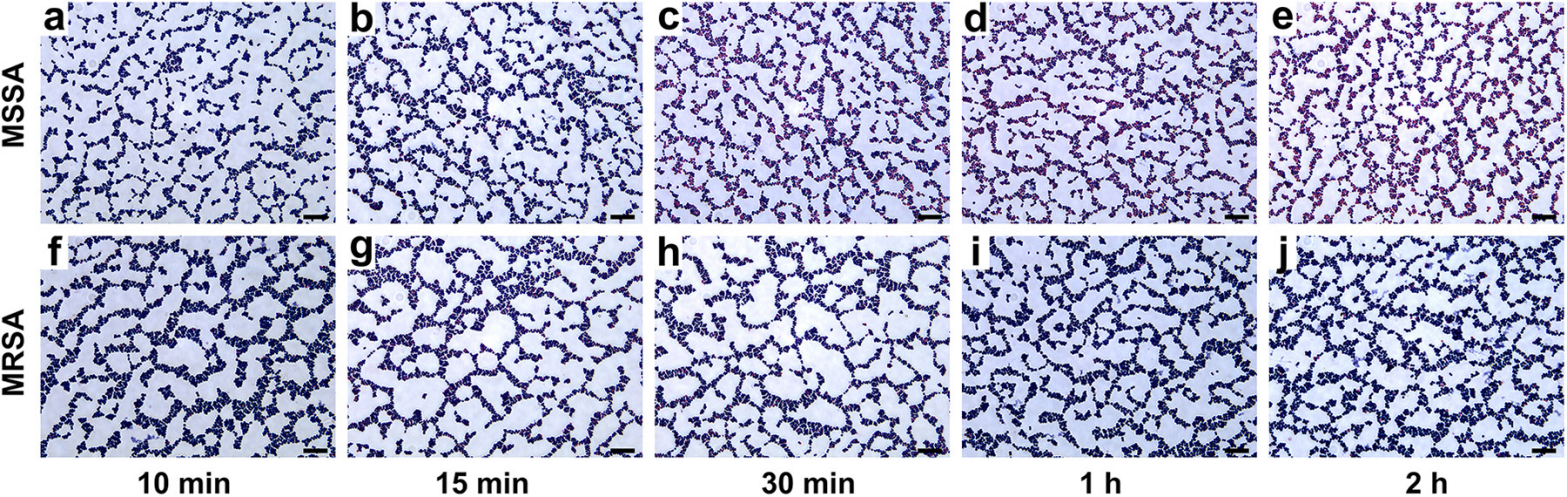
Microscopic images of MSSA strain 21B09710 (a to e) and MRSA strain 21B07569 (f to j) at 10 min, 15 min, 30 min, 1 h, and 2 h of incubation. Scale bars = 10 µm.

Figure 3 shows the microscope images of the staining of MSSA strain 21B09710 and MRSA strain 21B07569 incubated with 256 μg mL^−1^ oxacillin sodium salt for 10 min, 15 min, 30 min, 1 h, or 2 h. When the incubation time was 10 or 15 min, purple cocci accounted for the majority of MSSA and MRSA cells under the microscope (Fig. 3a and b and Fig. 3f and g), so MSSA and MRSA could not be distinguished. However, the proportion of pink cocci in MSSA gradually increased (Fig. 3c to e), whereas most MRSA cells were still stained purple (Fig. 3i and j), which clearly distinguished MSSA from MRSA. Therefore, the minimum time for bacteria to interact with oxacillin sodium salt was 30 min.

### Microscopic images of 50 clinical strains.

**FIG 4:**
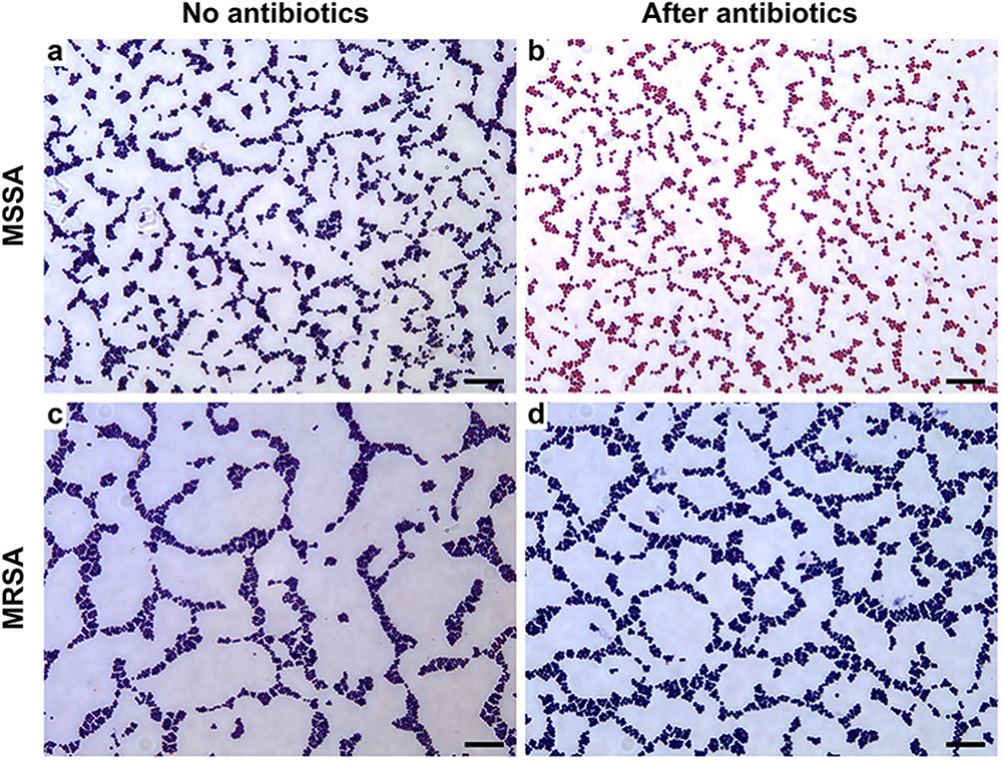
Microscopic images of MSSA strain 21W03255 (a and b) and MRSA strain 21R05422 (c and d) after Gram staining. (a and c) Staining results without oxacillin sodium salt; (b and d) staining results using oxacillin sodium salt. Scale bars = 10 µm.

Taking one MSSA strain and one MRSA strain as examples, Fig. 4a and b show the microscope images of strain 21W03255 without and with medication, and Fig. 4c and d show the microscope images of strain 21R05422 without and with antibiotics. Without oxacillin sodium salt after staining (Fig. 4a and c), the bacteria are stained purple. Compared to Fig. 4a, the proportion of pink cocci in Fig. 4b is significantly higher. Furthermore, compared to Fig. 4c, the bacteria in Fig. 4d are still stained purple. Therefore, strain 21W03255 was MSSA. Most of the bacteria in Fig. 4c and d were purple cocci, so strain 21R05422 was MRSA. The images of the remaining two parallel experiments are shown in Fig. S1 and S50 in the supplemental material. The images of the remaining 48 strains in three parallel experiments are shown in Fig. S2 to S49.

### Classification using MV analysis.

**FIG 5:**
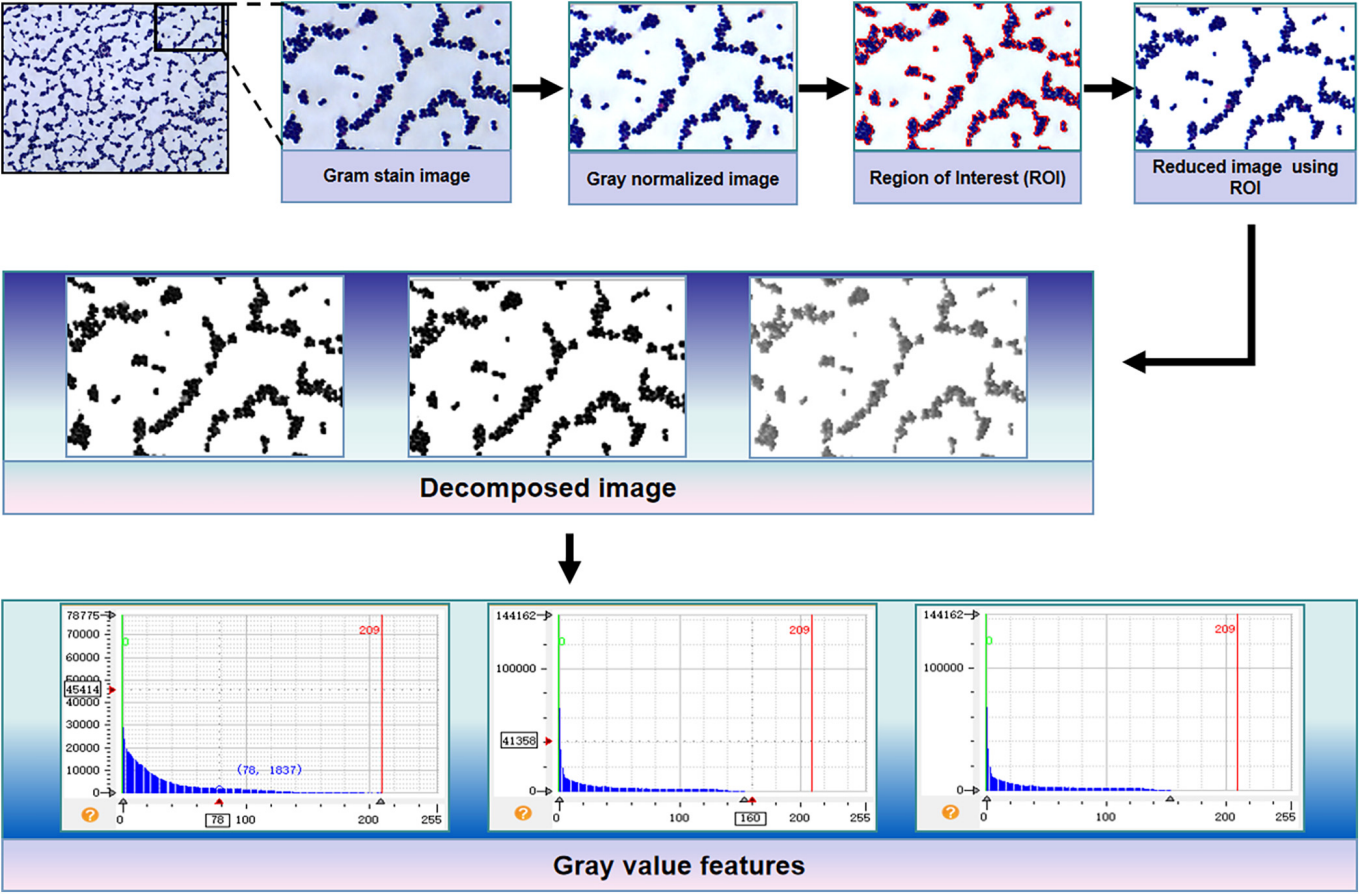
Procedure for preprocessing and feature extraction of staining images.

**FIG 6:**
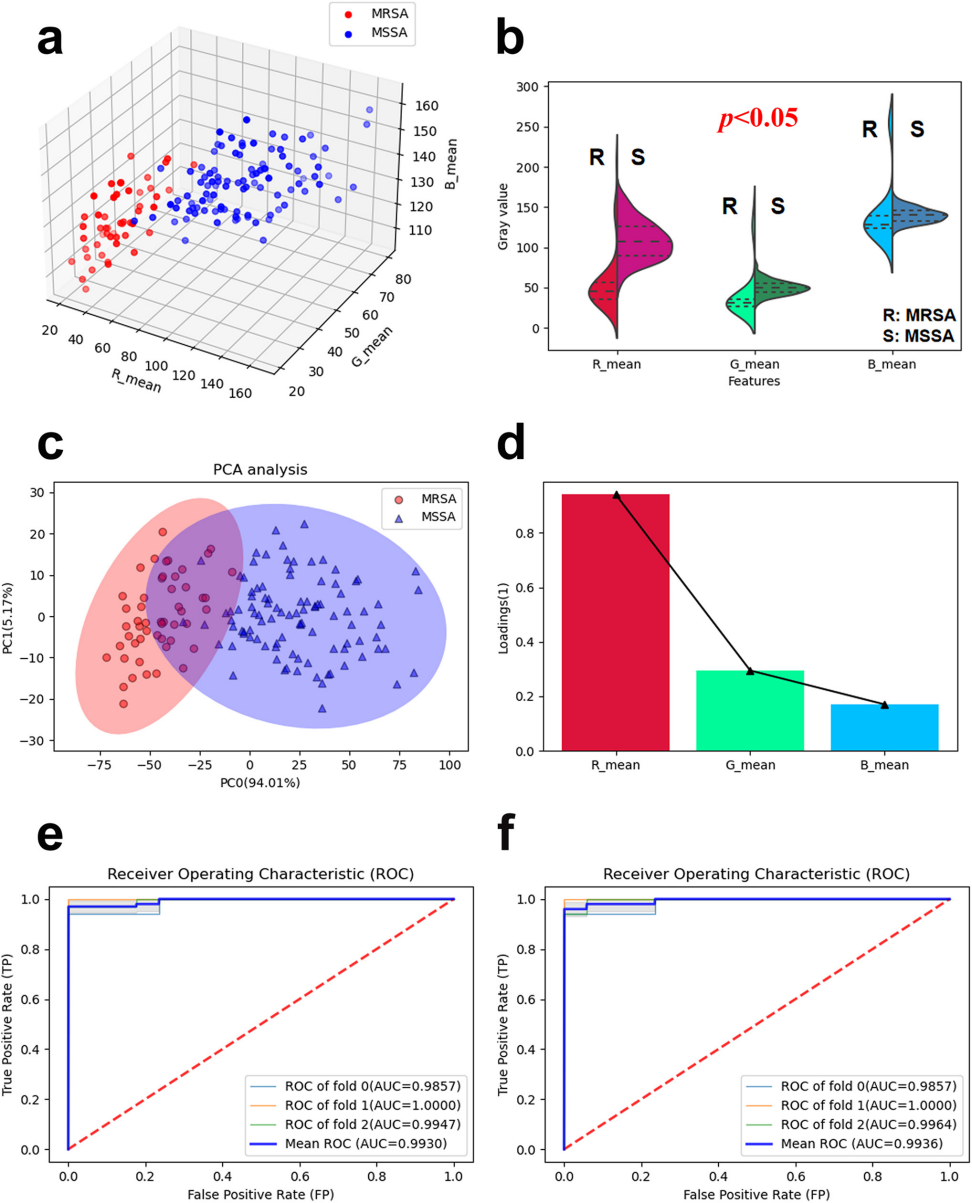
(a) 3D plot of the feature values of for the two classes; (b) violin plots of the feature values for the two classes. the *P* value is indicated on the top of the violin plot. The *P* values of comparisons these three features between the two classes are all <0.05. (c) PCA score plot of the data set; (d) loading plot for the first principal component of PCA; (e and f) receiver operating characteristic curves for screening of MSSA and MRSA strains through the LDA model (e) and ANN model (f). The areas under the mean ROC curve (AUC) were 0.9930 and 0.9936, respectively. A 3-fold cross-validation approach was employed.

For image preprocessing and feature extraction, the gray statistics-based method was used to preprocess and extract the color features of the bacterial region from the microscopic images, including the following steps (as shown in Fig. 5). (i) All of the staining images were stretched to the same grayscale (0 to 255) to generate a normalized image to eliminate the variation caused by the systematic or random variation (ambient brightness, camera parameters, etc.). (ii) The bacterial region of interest (ROI) was extracted using a binary threshold operator, which can segment the image using an automatically determined global threshold derived from the histogram of this image. (iii) The reduced image containing only bacterial areas was calculated as the intersection of the whole image domain with the ROI. The reduced color image was converted to red, green, and blue channel images, and the average gray value of the one-channel image was calculated (R_mean, G_mean, B_mean) to generate the feature values for further analysis. The feature values of each picture were displayed in a three-dimensional (3D) plot (Fig. 6a), in which each axis represents a feature value. Violin plots characterize the distribution of these three features of the two classes (MRSA and MSSA) (Fig. 6b). Notably, the R_mean feature (gray value in the red channel) showed a significant difference between the two classes, whereas the other two features gave a relatively small difference, which was confirmed in the principal-component analysis (PCA) loading plot (Fig. 6d). The PCA loading coefficient of the R_mean feature was obviously higher than the other two features. Notably, excellent separation was achieved using these three features in both PCA (Fig. 6c) and t-SNE (*t*-distributed stochastic neighbor embedding) analysis for the two classes (Fig. S51).

Based on these three features, we performed classification via two different supervised models: the linear discriminant analysis (LDA) model and the nonlinear artificial neural network (ANN) model. For parameter tuning and evaluation of generalization, data from 150 samples were included for model training, validation, and optimization using a 3-fold cross-validation. In particular, the data in the data set (including 150 staining images, one image for each strain, 3 experiments repeated, 50 strains total) were randomly divided into three folds: two folds were used for training and the remaining fold for validation, cycling this three times until all data were involved in training and testing. Specifically, the classifier consists of three layers: one input layer, one hidden layer (with 10 nodes), and one output layer. The solver for weight optimization is stochastic gradient descent (sgd), and the activation function for the hidden layer is the rectified linear unit (relu). We computed the average area under the curve (AUC) and accuracy of the data set during this process and repeated this multiple times with adjustment parameters until the best performance of the model was achieved. The optimized model was finally used for statistical analysis. The receiver operating characteristic (ROC) curves based on LDA and ANN are shown in Fig. 5e and f, respectively. The mean AUCs were 0.9930 and 0.9936 for LDA and ANN, respectively. The accuracies of these two models were 96.7% and 97.3% (Table 1), respectively. Furthermore, we confirmed that there was no overfitting for LDA and ANN classifier modeled by the extracted gray value features, based on the permutation test (Fig. S52). Both of the two learning models showed high classification performance. The results indicate that the performance of each of these models is fairly feasible.

**TABLE 1:** Performance of the classification models

Model	Accuracy	Sensitivity	Specificity	Precision	F1 score
LDA	0.9667	0.9596	0.9804	0.9899	0.9744
ANN	0.9733	0.9798	0.9608	0.9810	0.9798

## DISCUSSION

In this study, a simple and rapid method of identifying MRSA and MSSA based on the action of oxacillin sodium salt combined with Gram staining and MV analysis was reported. A number of clinical MRSA and MSSA strains were treated with oxacillin sodium salt, followed by Gram staining and microscopic examination. The gray statistics-based method was used to extract the color features of the microscope images of the Gram staining. The color change in MRSA and MSSA was detected by AI MV and discriminated by the LDA and ANN models. The MRSA-MSSA discrimination accuracies were 96.7% and 97.3%, respectively. This simple strategy improved the efficiency of detection and significantly shortened the time for detecting antibiotic resistance. The whole process could be completed within 1 h. In contrast to traditional ASTs, this process avoids overnight incubation.

This new technique could be used for other bacteria and may represent a new rapid method for clinically identifying antibiotic resistance, with emergency applications.

## MATERIALS AND METHODS

### Reagents and materials.

Tryptone soybean agar (TSA) and Luria-Bertani (LB) broth were purchased from Beijing Land Bridge Technology Co., Ltd. (Beijing, China). Oxacillin sodium salt was obtained from Shanghai Macklin Biochemical Technology Co., Ltd. (Shanghai, China). Crystal violet stain solution (1%), safranin solution, Gram’s iodine solution, and slides were obtained from Beijing Solarbio Science & Technology Co., Ltd. (Beijing, China). The Milli-Q deionized water system was obtained from Jingyuan Science & Technology Co., Ltd. (Hangzhou, China). The spectrophotometer, Heratherm, and bacterial incubator were obtained from Thermo Fisher Scientific (American). The centrifuge was purchased from Eppendorf (Germany). The Leica inverted microscope (DMi8) was obtained from Zhejiang Scientific Instruments & Materials I/E Co., Ltd. (Zhejiang, China).

### Bacterial strains.

S. aureus clinical isolates, including 33 strains of MSSA and 17 strains of MRSA, were collected from the Clinical Microbiology Laboratory at Peking Union Medical College Hospital (Beijing, China). The AST used the agar dilution method according to the recommendations of the Clinical and Laboratory Standards Institute (CLSI [USA]).

### Optimization of oxacillin sodium salt concentration.

The bacterial solution of MSSA and MRSA, with an optical density at 600 nm (OD_600_) of 0.7 to 0.8, was diluted to 10^7^ CFU·mL^−1^ using LB. The solution of oxacillin sodium salt (1,000 μg mL^−1^) was prepared by dissolving 1 mg oxacillin sodium salt in 1 mL of sterile water. The prepared solution was diluted step by step with sterile water to 512, 256, 128, 32, or 8 μg mL^−1^. We added 500 μL of the oxacillin sodium salt solutions at different concentrations into 500 μL of 10^7^ CFU·mL^−1^ MSSA and MRSA bacterial solutions. After incubation at 37°C for 30 min at 1,250 rpm, the mixture was centrifuged at 10,000 rpm for 2 min and then washed twice with 100 μL of sterile water. We added 20 μL sterile water to the sediment, transferred it to a slide, dried it at 40°C, and performed Gram staining with microscopic examination.

### Optimization of incubation time for oxacillin sodium salt and bacteria.

The oxacillin sodium salt solution (256 μg mL^−1^, 500 μL) was added to each of the 10 centrifuge tubes containing the MSSA (10^7^ CFU·mL^−1^, 500 μL) and MRSA (10^7^ CFU·mL^−1^, 500 μL) solutions at the same time, but incubated separately at 1,250 rpm at 37°C for 10 min, 15 min, 30 min, 1 h, and 2 h. After incubation, the mixture was centrifuged at 10,000 rpm for 2 min and washed twice with 100 μL of sterile water. We added 20 μL sterile water to the sediment, transferred it to a slide, dried the slide at 40°C, and performed Gram staining with microscopic examination.

### Screening of 50 clinical strains of MSSA and MRSA.

Under optimal conditions, the oxacillin sodium salt solution (256 μg mL^−1^, 500 μL) was added to 50 bacterial solutions (10^7^ CFU·mL^−1^, 500 μL) and incubated at 37°C for 30 min at 1,250 rpm. After incubation, the supernatant was removed by centrifugation at 10,000 rpm for 2 min, and then 100 μL of sterile water was added for washing twice. We added 20 μL of sterile water to the precipitate, transferred it to a slide, dried the slide at 40°C, and then performed the staining with microscopic examination. Through three parallel experiments, the obtained images were used for MV analysis.

### MV analysis.

MV analysis of the staining images included preprocessing and feature extraction, model optimization, and validation. For preprocessing and feature extraction, all of the staining images were normalized by grayscale stretching to eliminate the calculation error caused by the brightness of different pictures. Grayscale-based segmentation technology was employed to extract the region of interest (ROI, i.e., bacterial region) in the images, and then the normalized image of the ROI was reduced. Grayscale features of the reduced image were counted as the feature data for further analysis. Image analyses during this procedure were conducted on HALCON (v.12.0; MVTec Software GmbH, Germany). For model optimization and validation, the unsupervised analyses (principal-component analysis [PCA] and *t*-distributed stochastic neighbor embedding [t-SNE]) were conducted to visualize the feature data. Both linear and nonlinear classification models were attempted for classification. In particular, the simple and reliable linear discriminant analysis (LDA) and more complicated learning-based artificial neural network (ANN) were both included for model construction. The receiver operating characteristic (ROC), area under the curve (AUC), and performance metrics, including accuracy, sensitivity, specificity, precision, and F1 score, were calculated to optimize and validate the model during a cross-validation procedure. Student's *t* test was used to calculate the *P* value for statistical demonstration. A permutation test was employed to test whether the established model is overfitting. The above analysis was performed with homemade code in Python (version 3.8), using scikit-learn, seaborn packages, etc.
