# Supplementary material for: Simple and Rapid Discrimination of Methicillin-Resistant Staphylococcus aureus Based on Gram Staining and Machine Vision
Source: Microbiol Spectr. 2023 Jul 3;11(4):e05282-22. doi: 10.1128/spectrum.05282-22 (PMC10433844; doi:10.1128/spectrum.05282-22)
Supplement: Supplemental file 1 — Supplemental material. Download spectrum.05282-22-s0001.pdf, PDF file, 11.8 MB [file spectrum.05282-22-s0001.pdf]

Supplementary Material

Simple and Rapid Discrimination of Methicillin-Resistant *Staphylococcus aureus* Based on Gram Stain and Machine Vision

Menghuan Yu,[a,b] Haimei Shi,[a,b] Hao Shen,[a,b] Li Zhang,[c] Xueqin Chen,[a] Jianhua Zhu,[a] Guoqing Qian,[a] Bin Feng,[a,b*] Shaoning Yu[a,b*]

[a]Department of Intensive Care Unit, The First Affiliated Hospital of Ningbo University, Ningbo University, Ningbo, Zhejiang, China.

[b]Institute of Mass Spectrometry, School of Material Science and Chemical Engineering, Ningbo University, Ningbo, Zhejiang, China.

[c]Department of Clinical Lab, Peking Union Medical College Hospital, Peking Union Medical College & Chinese Academy Medical Science, Beijing, China.

Address correspondence to Bin Feng, fengbin@nbu.edu.cn; Shaoning Yu, yushaoning@nbu.edu.cn.

Three parallel experiments with the staining results for 50 clinical strains and t-SNE analysis are shown in Figure S1 to S51:

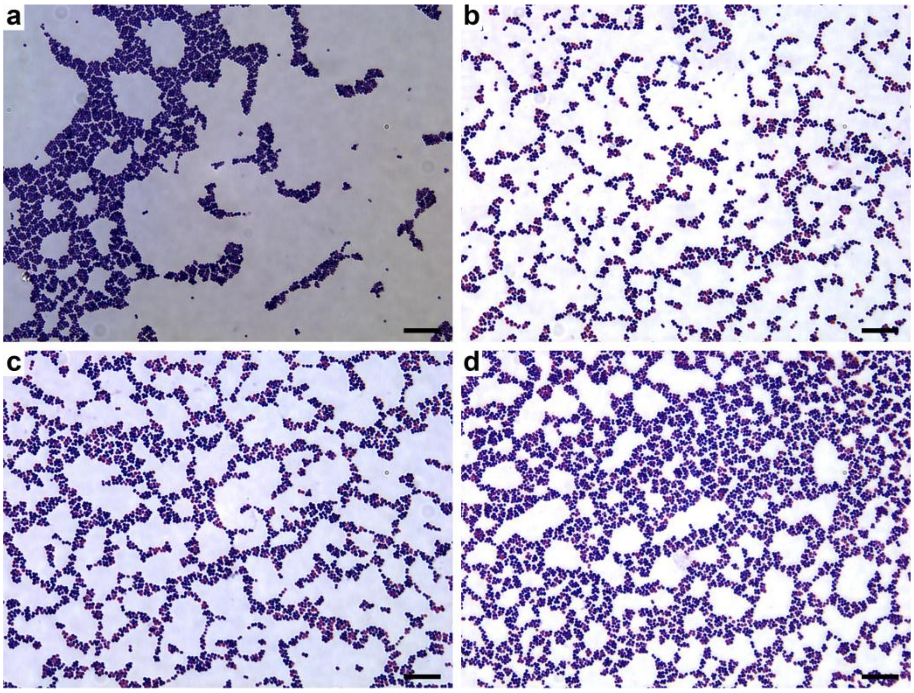

Figure S1 Microscopic images of MRSA strain 21B03641 after Gram staining. (a) Staining results without oxacillin sodium salt. (b-d) Three parallel experiments using oxacillin sodium salt. Scale bars = 10 μm.

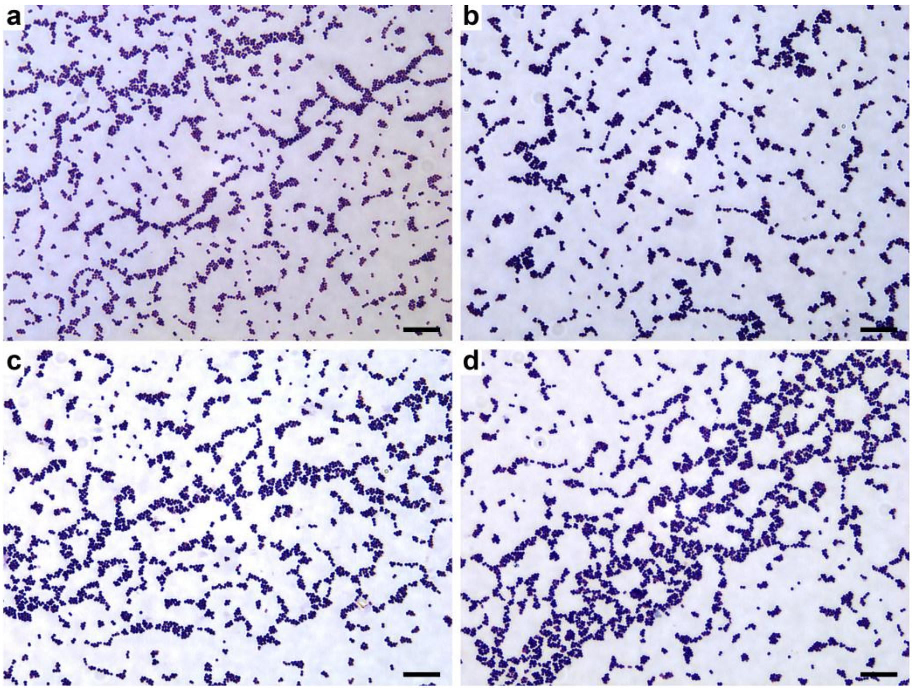

Figure S2 Microscopic images of MRSA strain 21B06749 after Gram staining. (a) Staining results without oxacillin sodium salt. (b-d) Three parallel experiments using oxacillin sodium salt. Scale bars = 10 μm.

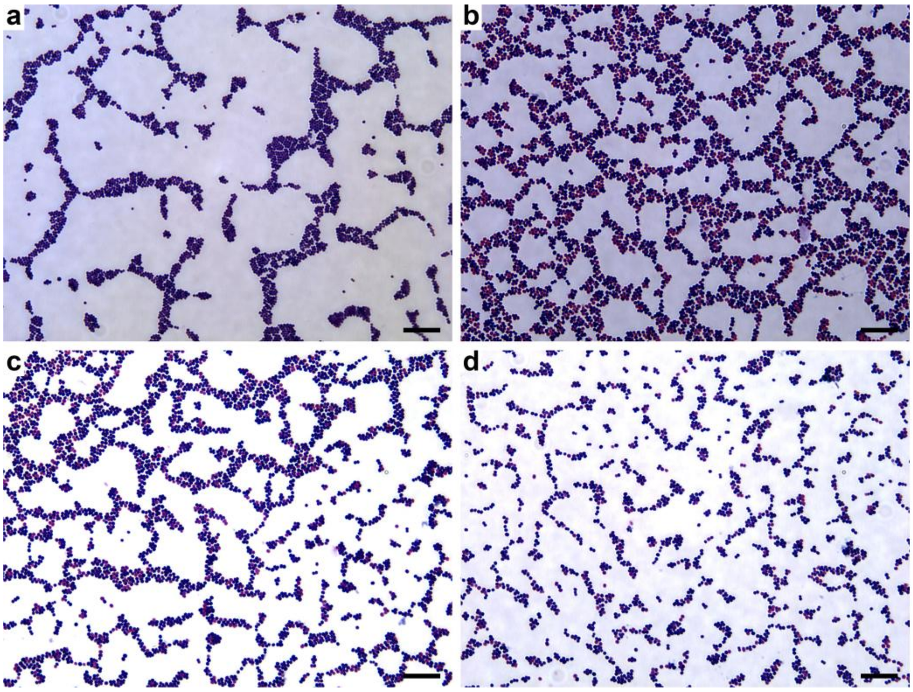

Figure S3 Microscopic images of MRSA strain 21B07044 after Gram staining. (a) Staining results without oxacillin sodium salt. (b-d) Three parallel experiments using oxacillin sodium salt. Scale bars = 10 μm.

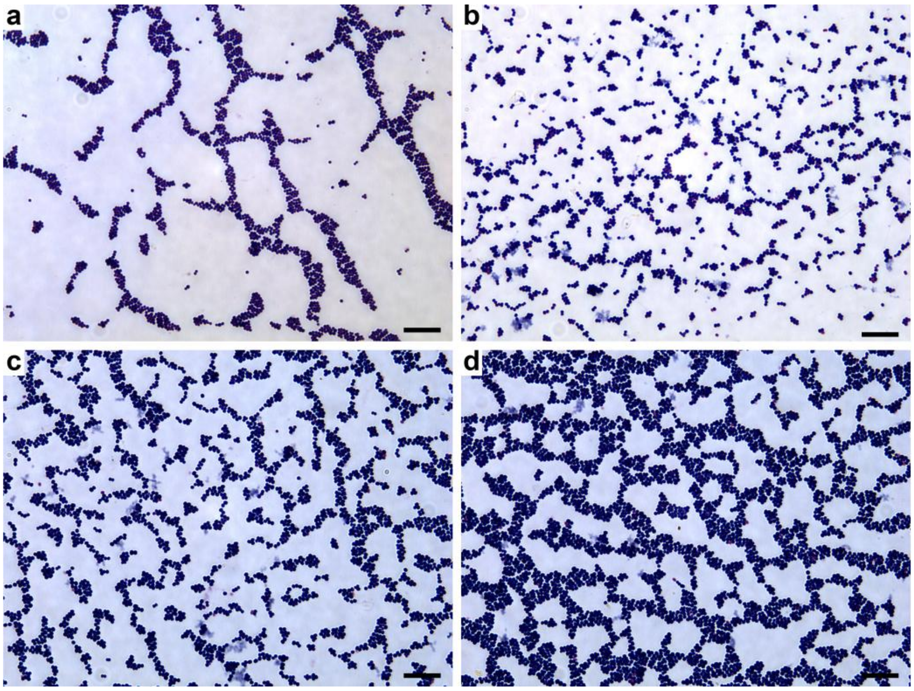

Figure S4 Microscopic images of MRSA strain 21B07569 after Gram staining. (a) Staining results without oxacillin sodium salt. (b-d) Three parallel experiments using oxacillin sodium salt. Scale bars = 10 μm.

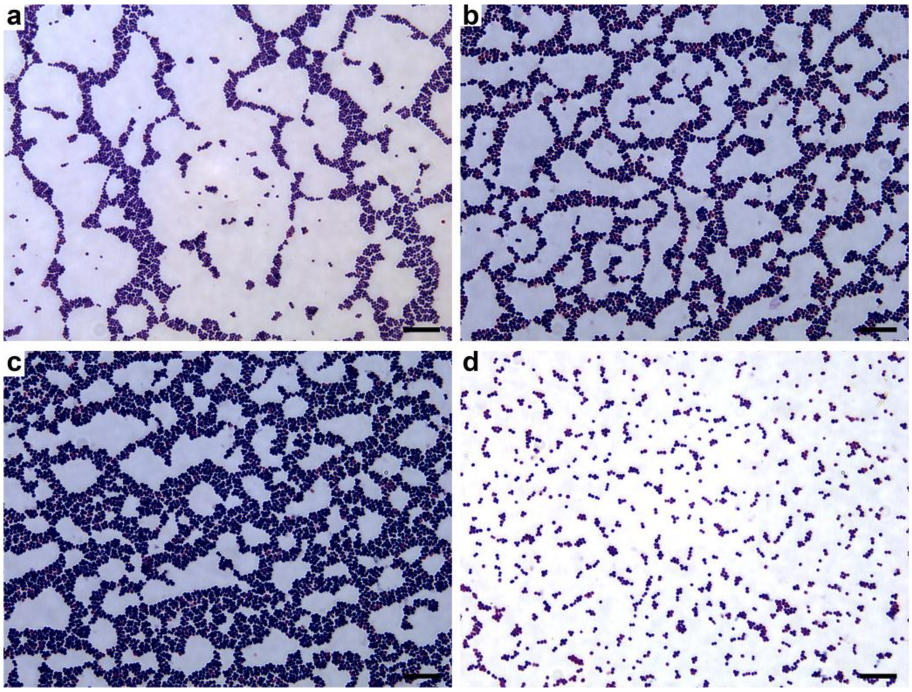

Figure S5 Microscopic images of MRSA strain 21B08249 after Gram staining. (a) Staining results without oxacillin sodium salt. (b-d) Three parallel experiments using oxacillin sodium salt. Scale bars = 10 μm.

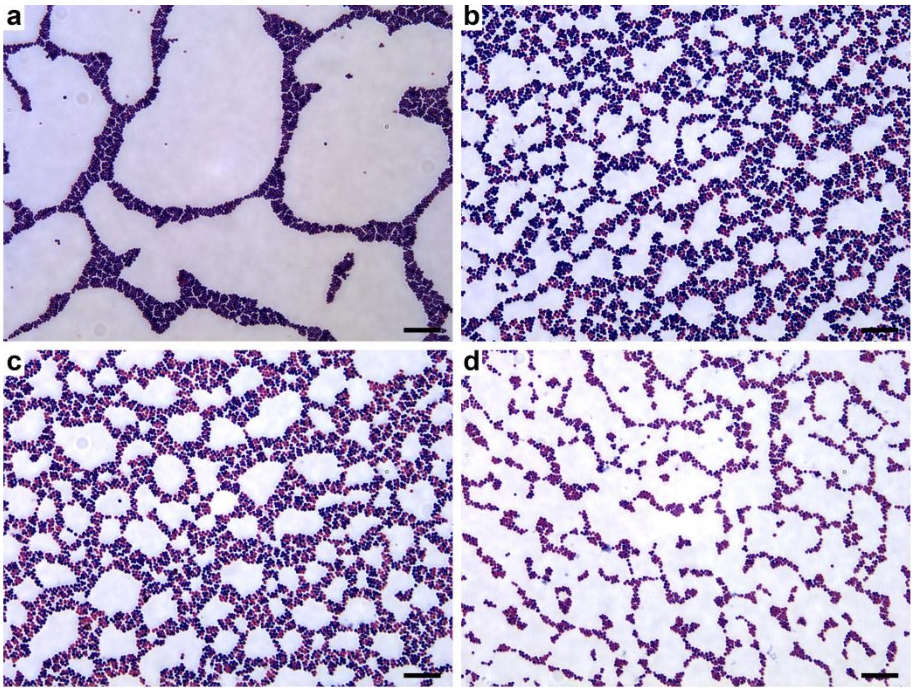

Figure S6 Microscopic images of MSSA strain 21B09565 after Gram staining. (a) Staining results without oxacillin sodium salt. (b-d) Three parallel experiments using oxacillin sodium salt. Scale bars = 10 μm.

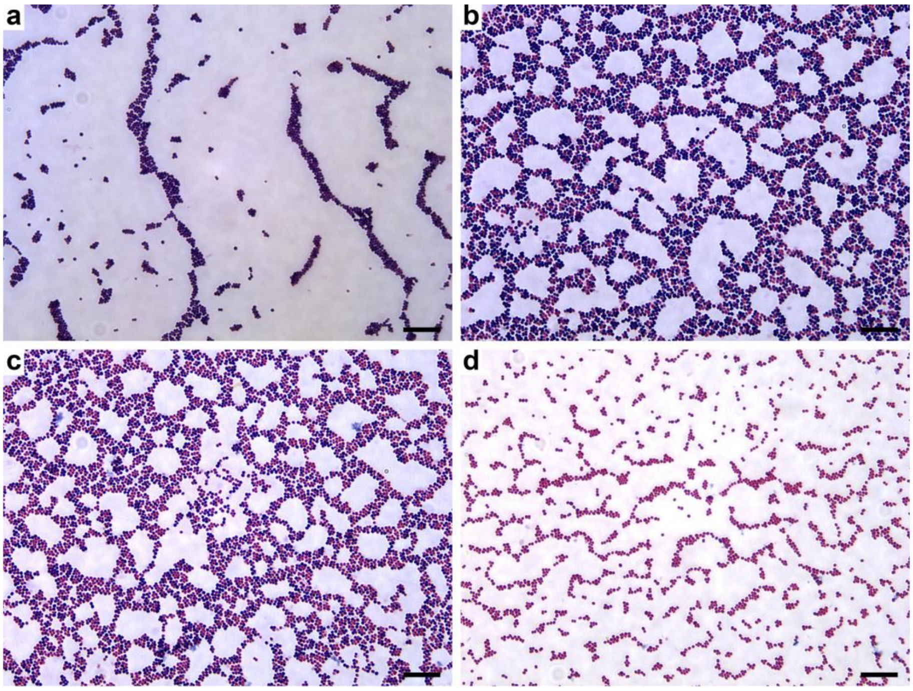

Figure S7 Microscopic images of MSSA strain 21B09625 after Gram staining. (a) Staining results without oxacillin sodium salt. (b-d) Three parallel experiments using oxacillin sodium salt. Scale bars = 10 μm.

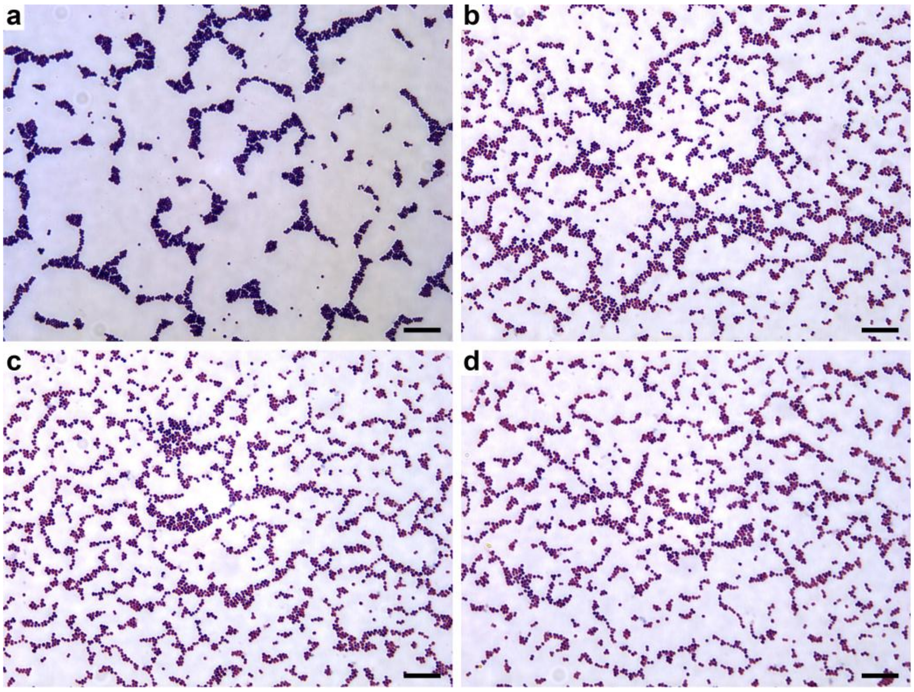

Figure S8 Microscopic images of MSSA strain 21B09710 after Gram staining. (a) Staining results without oxacillin sodium salt. (b-d) Three parallel experiments using oxacillin sodium salt. Scale bars = 10 μm.

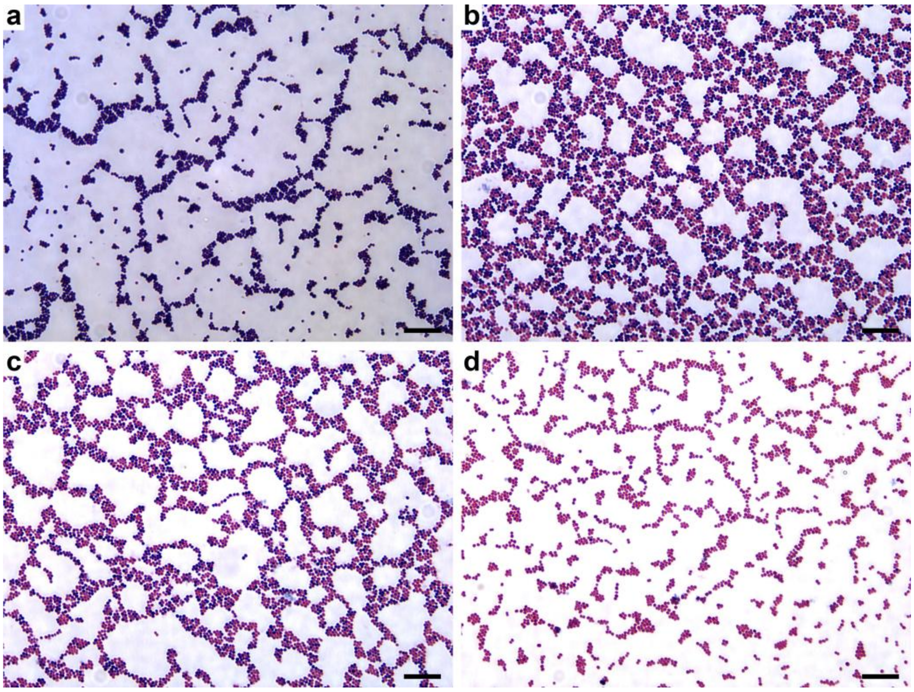

Figure S9 Microscopic images of MSSA strain 21B09730 after Gram staining. (a) Staining results without oxacillin sodium salt. (b-d) Three parallel experiments using oxacillin sodium salt. Scale bars = 10 μm.

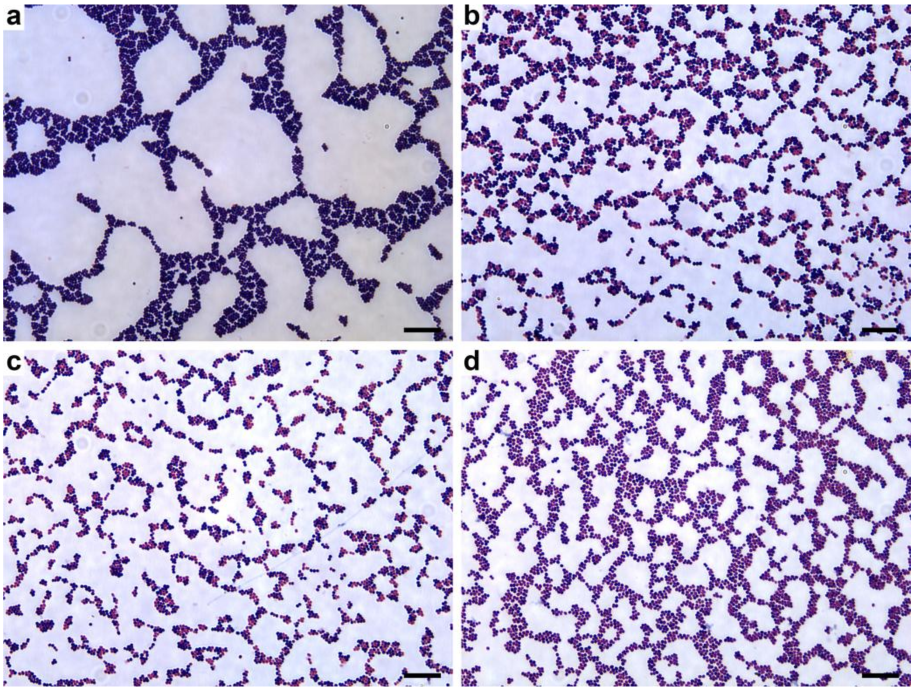

Figure S10 Microscopic images of MSSA strain 21B09791 after Gram staining. (a) Staining results without oxacillin sodium salt. (b-d) Three parallel experiments using oxacillin sodium salt. Scale bars = 10 μm.

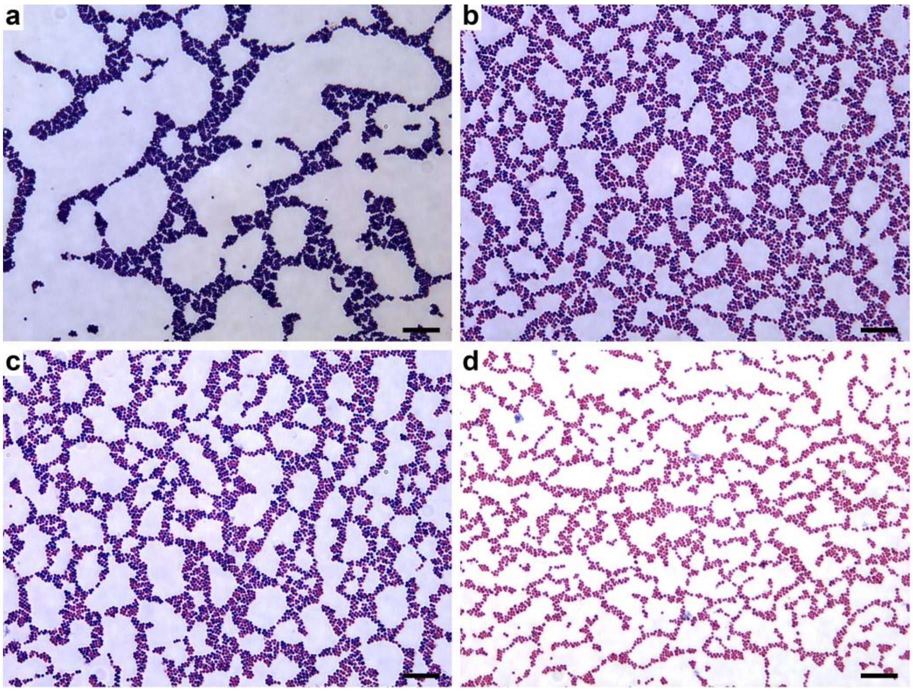

Figure S11 Microscopic images of MSSA strain 21B11043 after Gram staining. (a) Staining results without oxacillin sodium salt. (b-d) Three parallel experiments using oxacillin sodium salt. Scale bars = 10 μm.

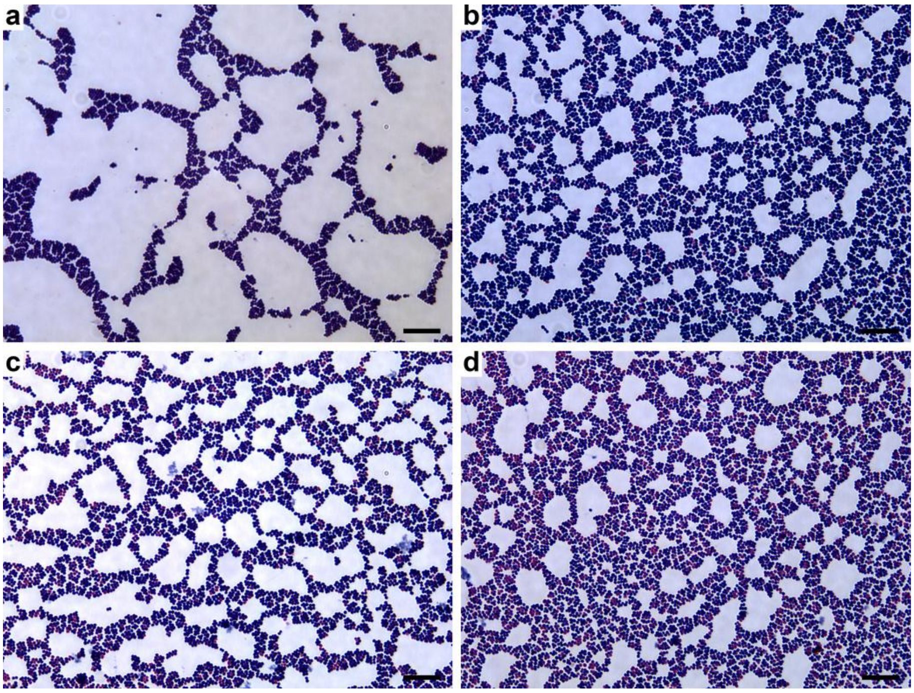

Figure S12 Microscopic images of MRSA strain 21B11183 after Gram staining. (a) Staining results without oxacillin sodium salt. (b-d) Three parallel experiments using oxacillin sodium salt. Scale bars = 10 μm.

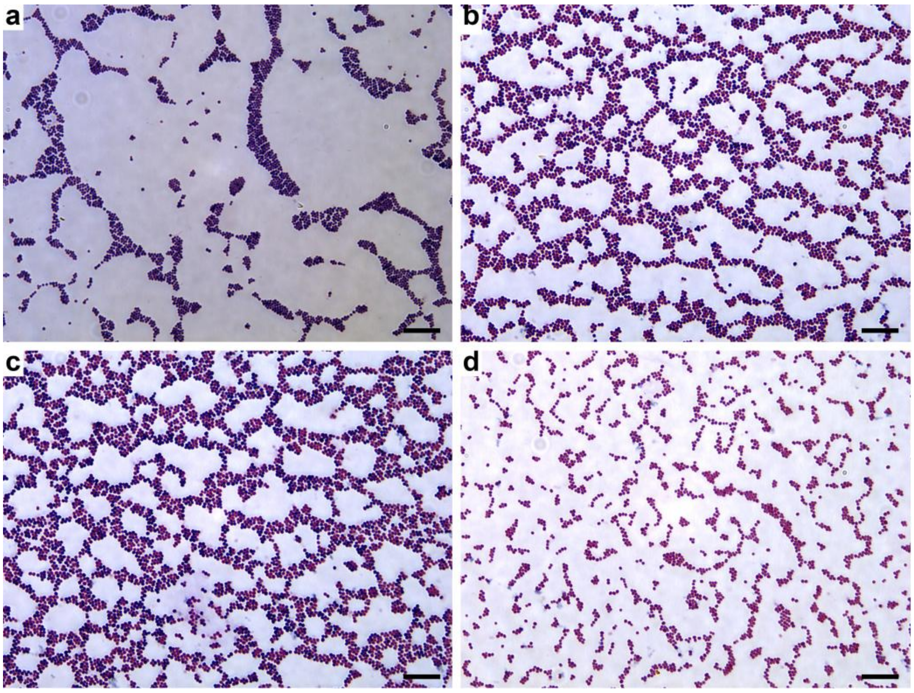

Figure S13 Microscopic images of MSSA strain 21B11780 after Gram staining. (a) Staining results without oxacillin sodium salt. (b-d) Three parallel experiments using oxacillin sodium salt. Scale bars = 10 μm.

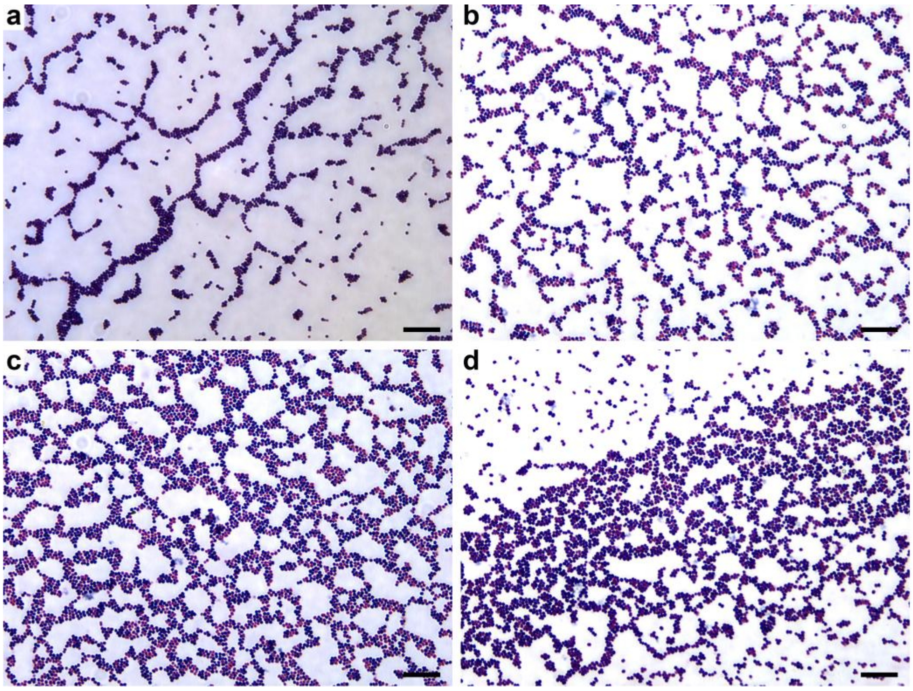

Figure S14 Microscopic images of MSSA strain 21B11864 after Gram staining. (a) Staining results without oxacillin sodium salt. (b-d) Three parallel experiments using oxacillin sodium salt. Scale bars = 10 μm.

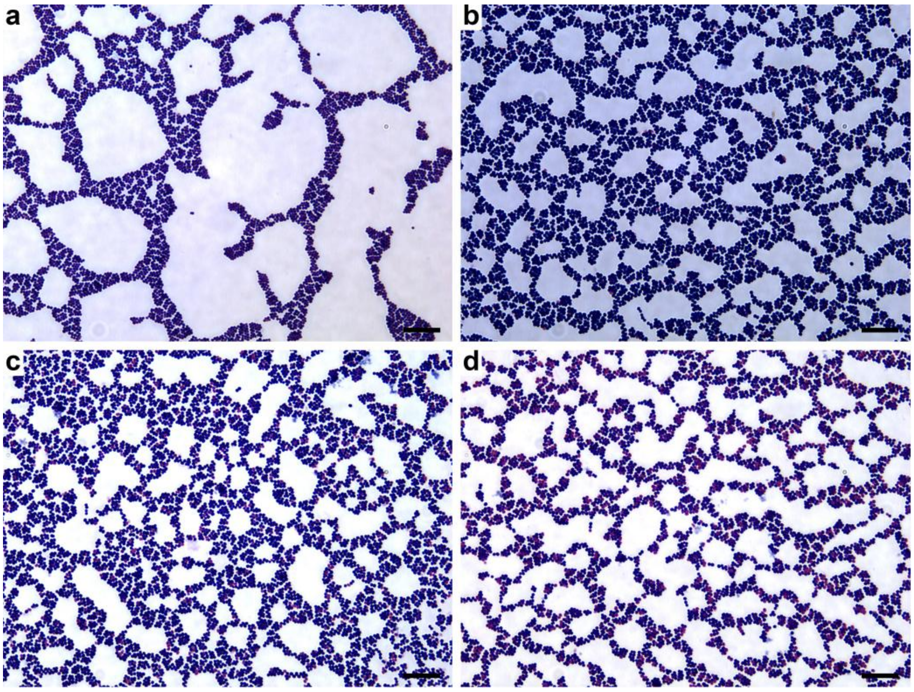

Figure S15 Microscopic images of MRSA strain 21C01158 after Gram staining. (a) Staining results without oxacillin sodium salt. (b-d) Three parallel experiments using oxacillin sodium salt. Scale bars = 10 μm.

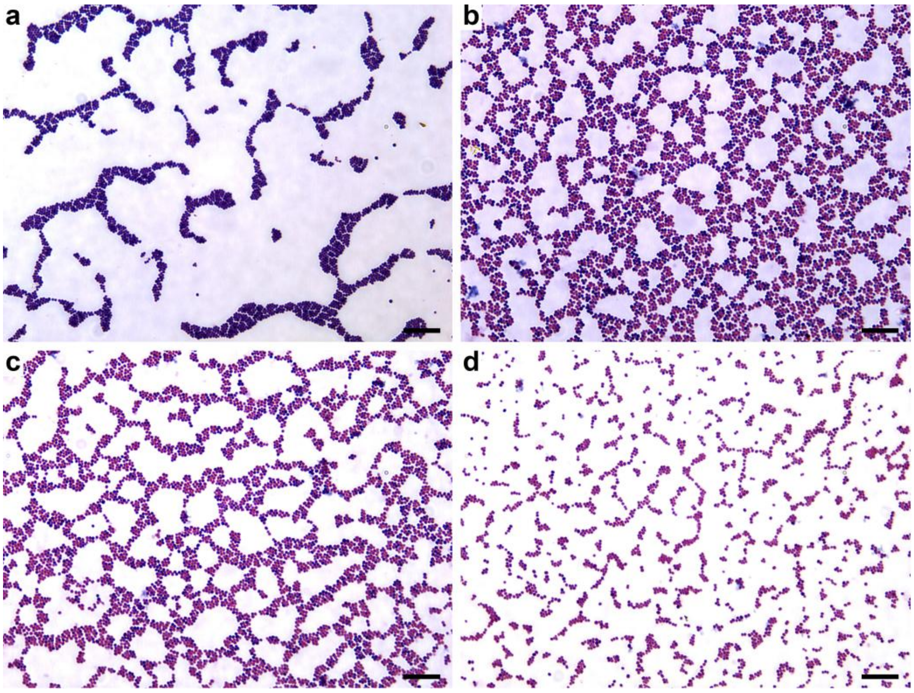

Figure S16 Microscopic images of MSSA strain 21C01161 after Gram staining. (a) Staining results without oxacillin sodium salt. (b-d) Three parallel experiments using oxacillin sodium salt. Scale bars = 10 μm.

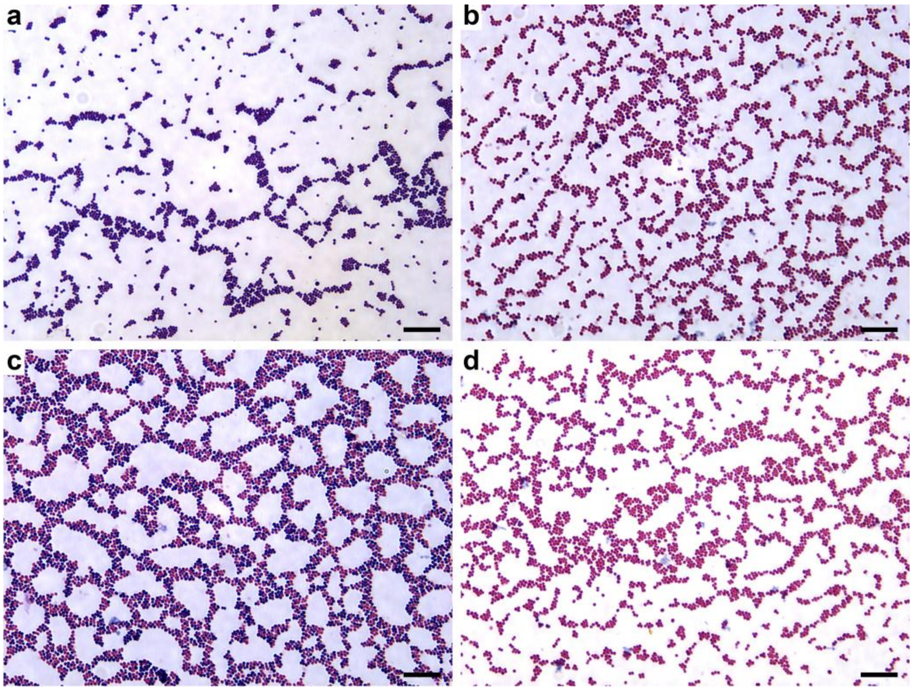

Figure S17 Microscopic images of MSSA strain 21C01222 after Gram staining. (a) Staining results without oxacillin sodium salt. (b-d) Three parallel experiments using oxacillin sodium salt. Scale bars = 10 μm.

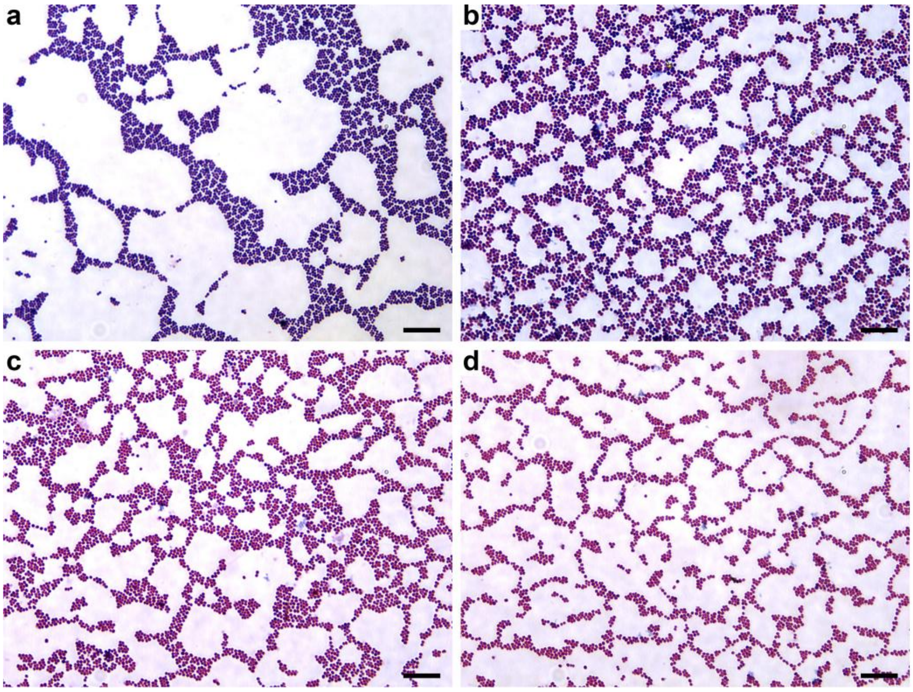

Figure S18 Microscopic images of MSSA strain 21C01383 after Gram staining. (a) Staining results without oxacillin sodium salt. (b-d) Three parallel experiments using oxacillin sodium salt. Scale bars = 10 μm.

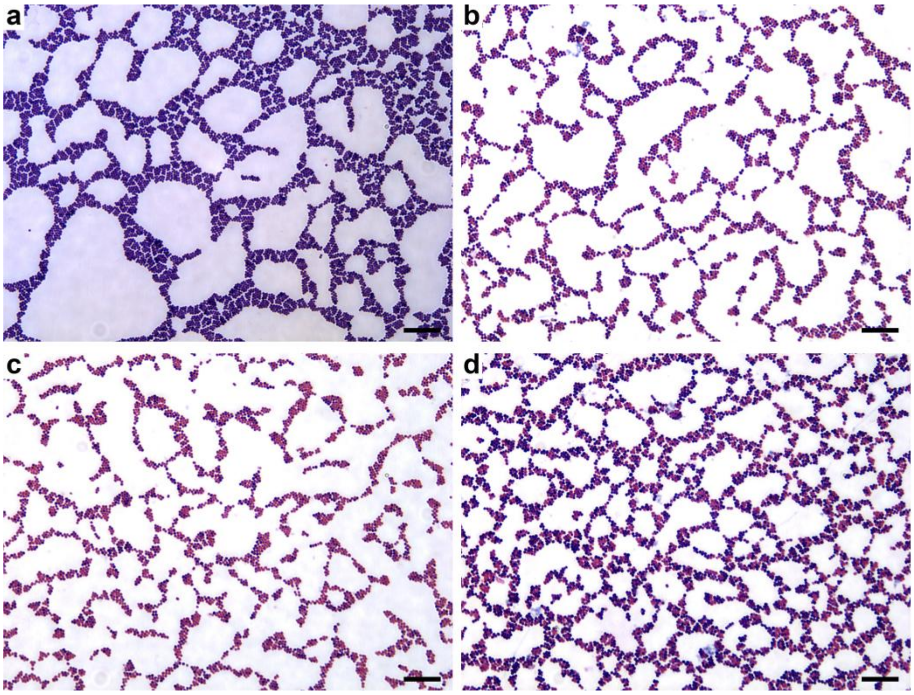

Figure S19 Microscopic images of MSSA strain 21R04907 after Gram staining. (a) Staining results without oxacillin sodium salt. (b-d) Three parallel experiments using oxacillin sodium salt. Scale bars = 10 μm.

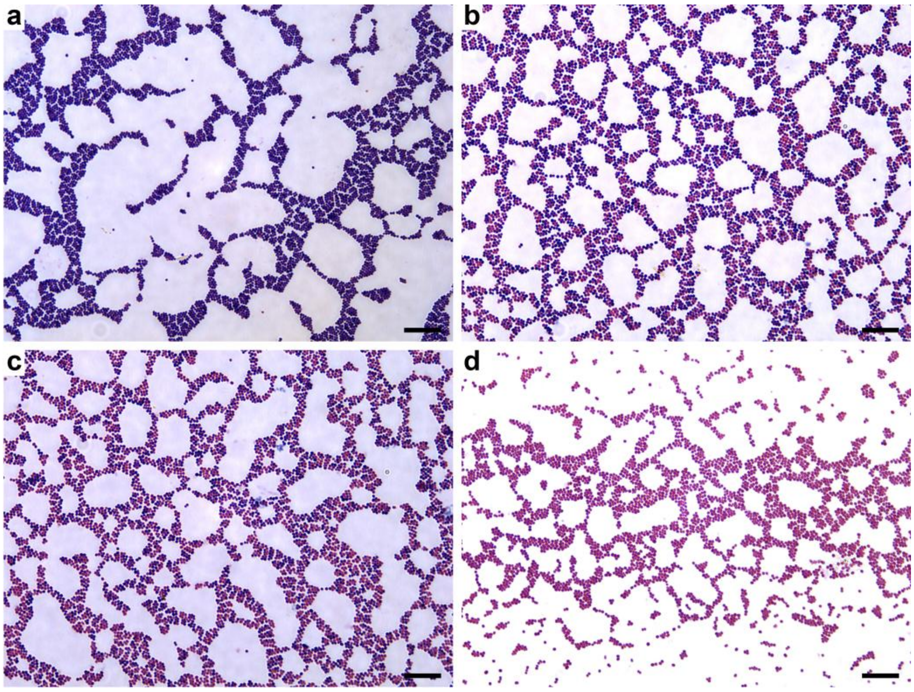

Figure S20 Microscopic images of MSSA strain 21R04987 after Gram staining. (a) Staining results without oxacillin sodium salt. (b-d) Three parallel experiments of staining results using oxacillin sodium salt. Scale bars are 10 μm.

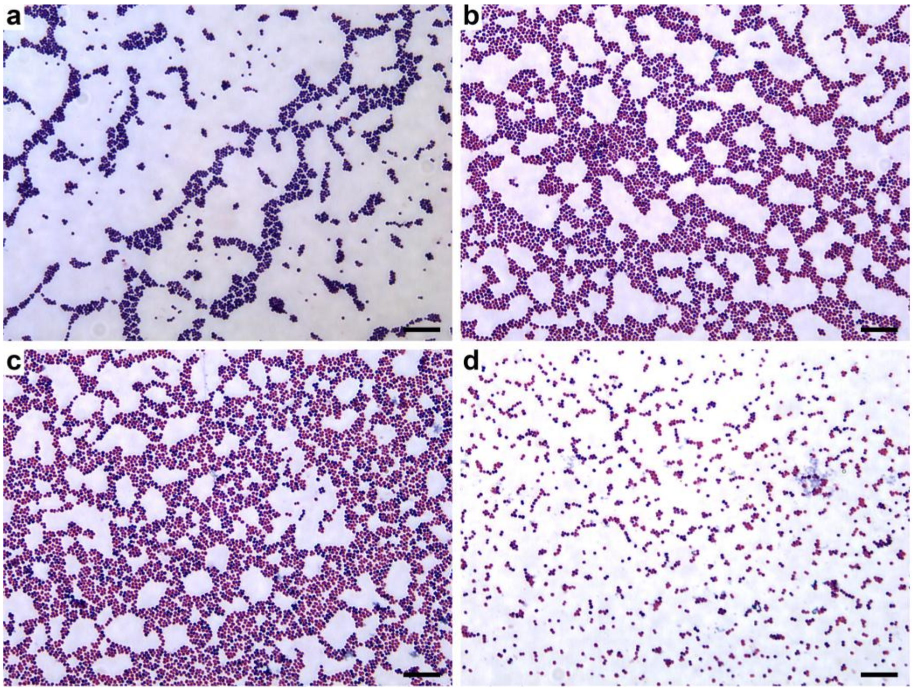

Figure S21 Microscopic images of MSSA strain 21R05171 after Gram staining. (a) Staining results without oxacillin sodium salt. (b-d) Three parallel experiments using oxacillin sodium salt. Scale bars = 10 μm.

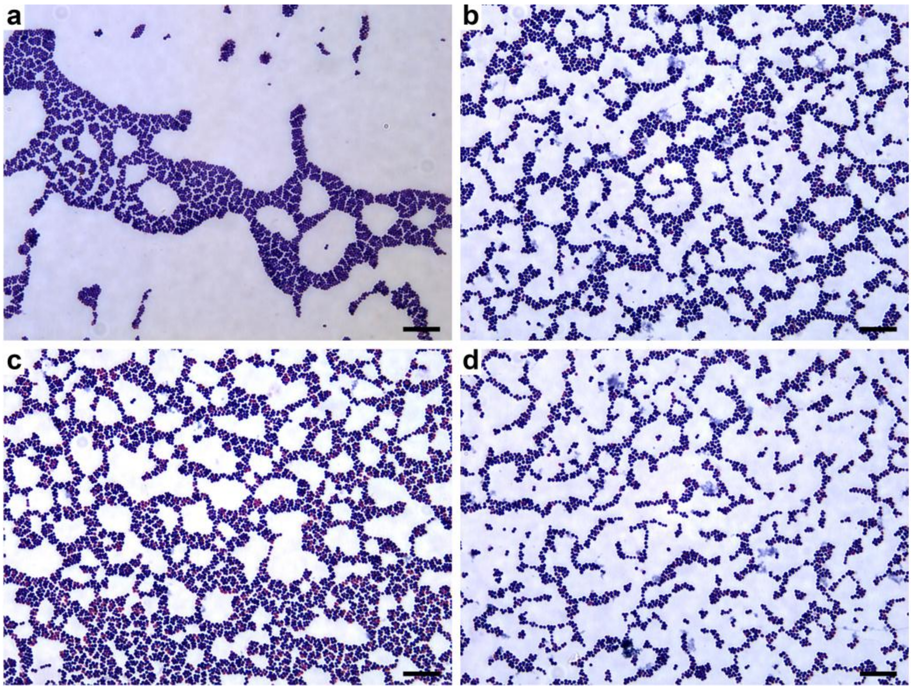

Figure S22 Microscopic images of MRSA strain 21R05220 after Gram staining. (a) Staining results without oxacillin sodium salt. (b-d) Three parallel experiments using oxacillin sodium salt. Scale bars = 10 µm.

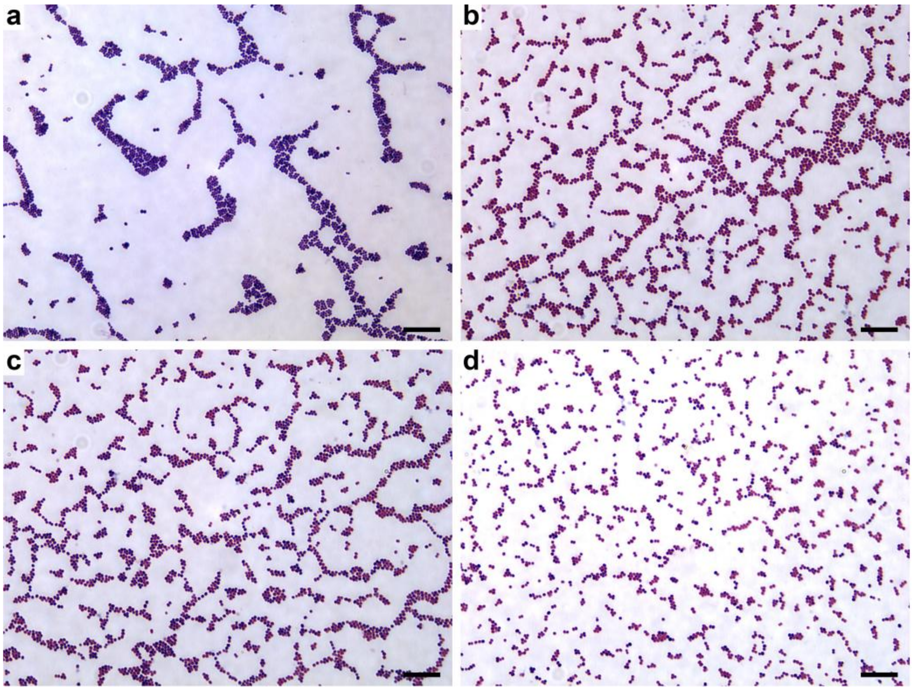

Figure S23 Microscopic images of MSSA strain 21R05221 after Gram staining. (a) Staining results without oxacillin sodium salt. (b-d) Three parallel experiments using oxacillin sodium salt. Scale bars = 10 µm.

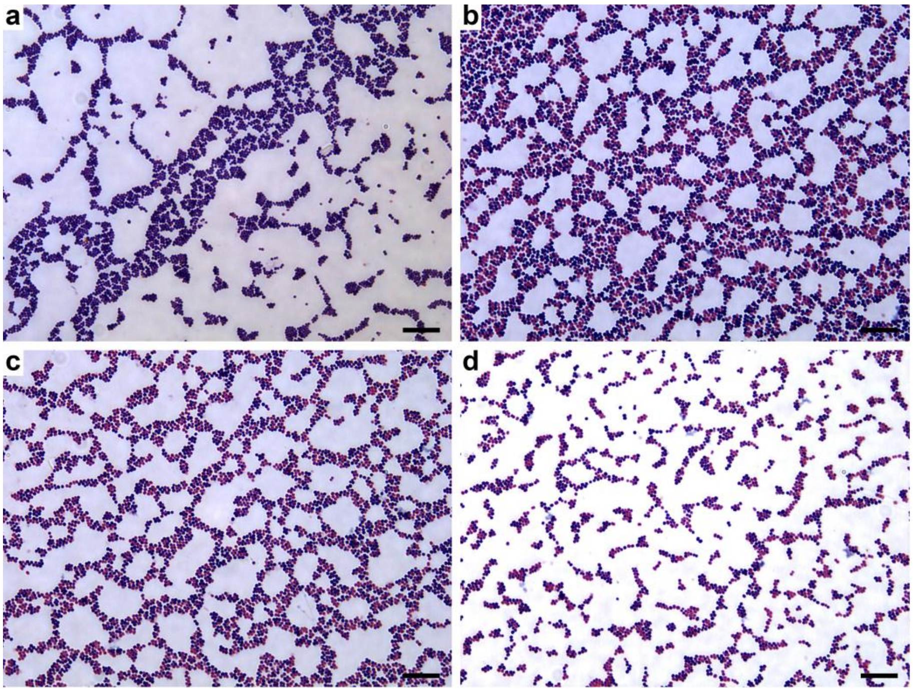

Figure S24 Microscopic images of MSSA strain 21R05288 after Gram staining. (a) Staining results without oxacillin sodium salt. (b-d) Three parallel experiments using oxacillin sodium salt. Scale bars = 10 μm.

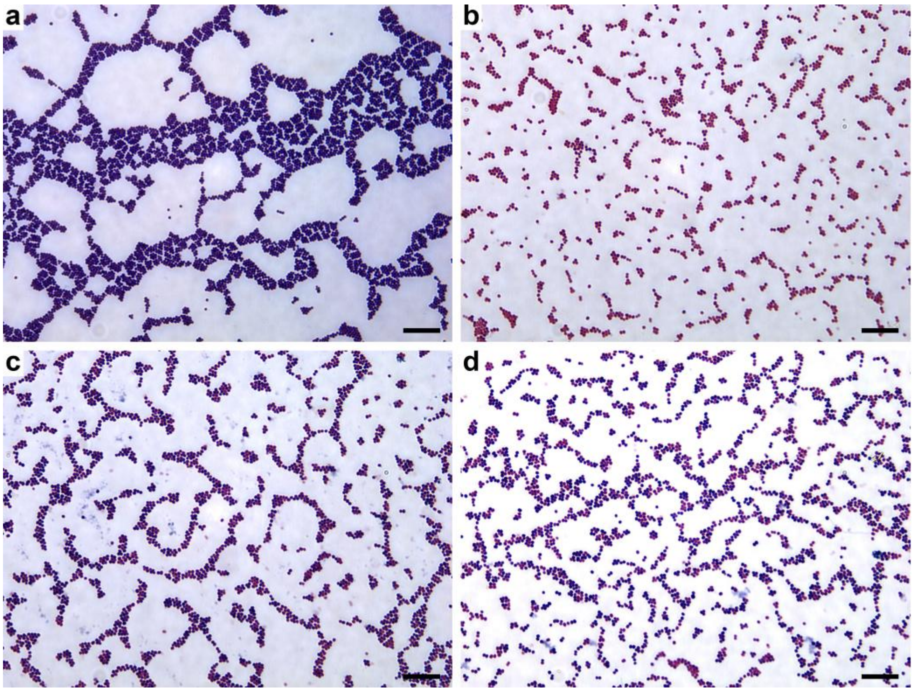

Figure S25 Microscopic images of MSSA strain 21R05322 after Gram staining. (a) Staining results without oxacillin sodium salt. (b-d) Three parallel experiments using oxacillin sodium salt. Scale bars = 10 μm.

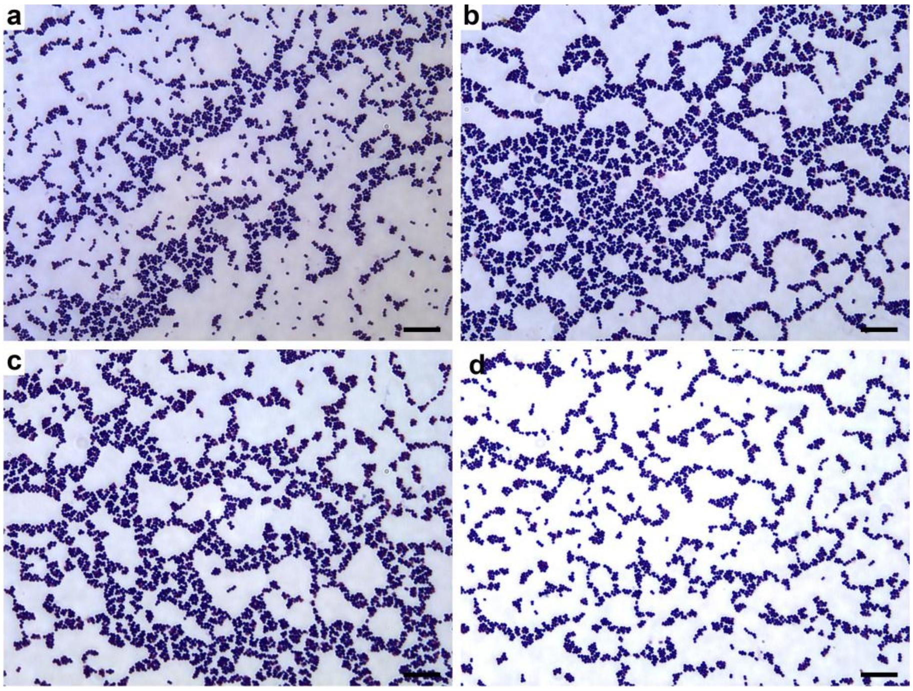

Figure S26 Microscopic images of MRSA strain 21R05333 after Gram staining. (a) Staining results without oxacillin sodium salt. (b-d) Three parallel experiments using oxacillin sodium salt. Scale bars = 10 μm.

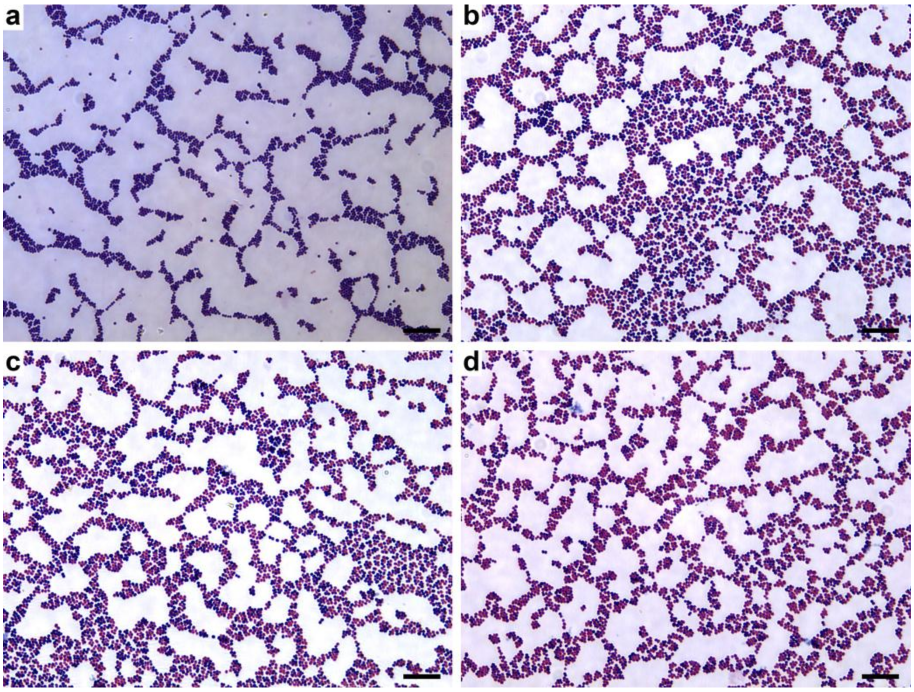

Figure S27 Microscopic images of MSSA strain 21R05342 after Gram staining. (a) Staining results without oxacillin sodium salt. (b-d) Three parallel experiments using oxacillin sodium salt. Scale bars = 10 μm.

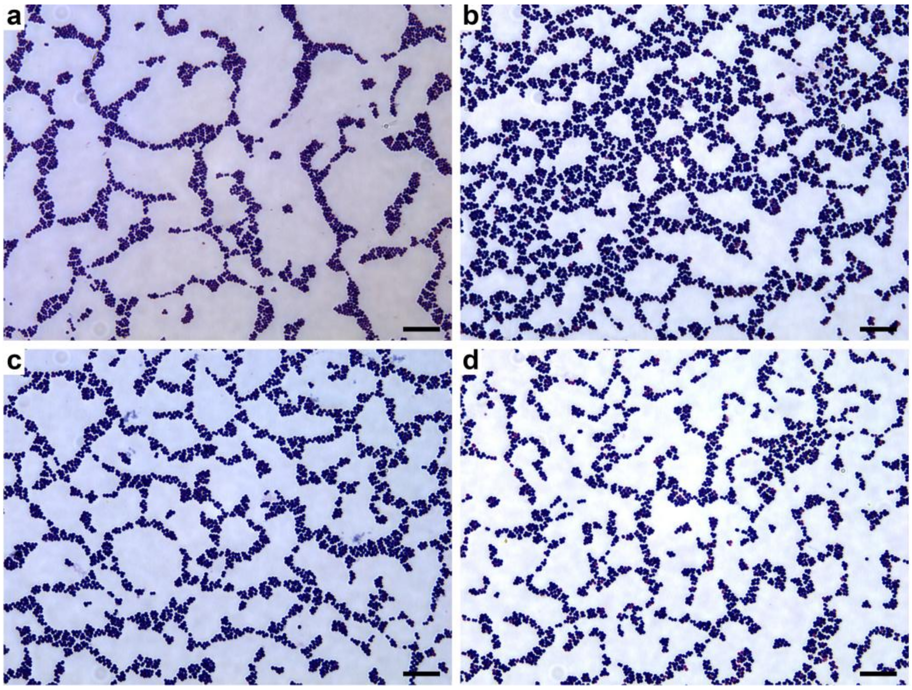

Figure S28 Microscopic images of MSSA strain 21R05422 after Gram staining. (a) Staining results without oxacillin sodium salt. (b-d) Three parallel experiments using oxacillin sodium salt. Scale bars = 10 μm.

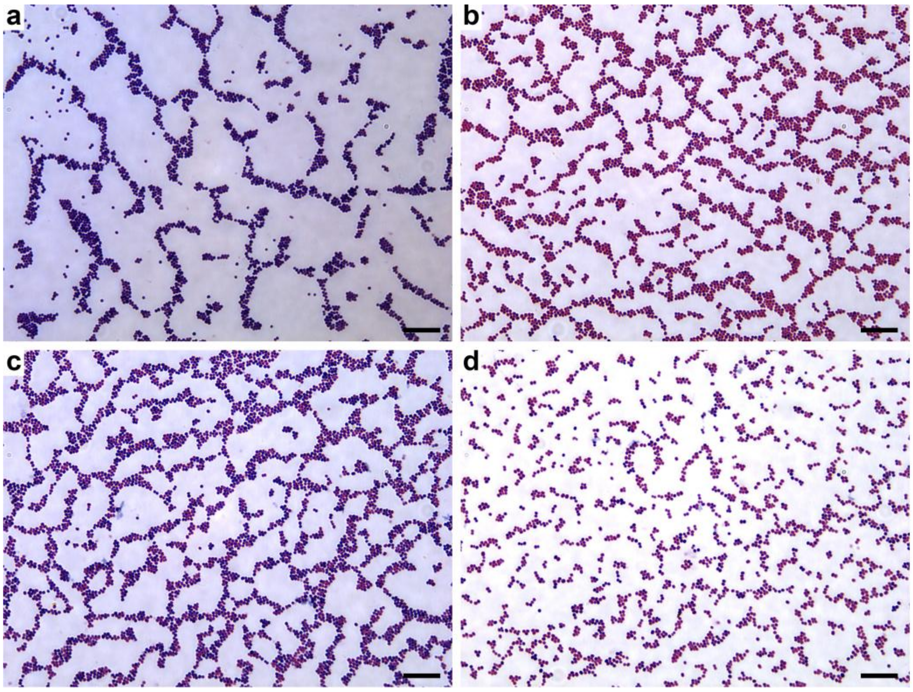

Figure S29 Microscopic images of MSSA strain 21R05646 after Gram staining. (a) Staining results without oxacillin sodium salt. (b-d) Three parallel experiments using oxacillin sodium salt. Scale bars = 10 μm.

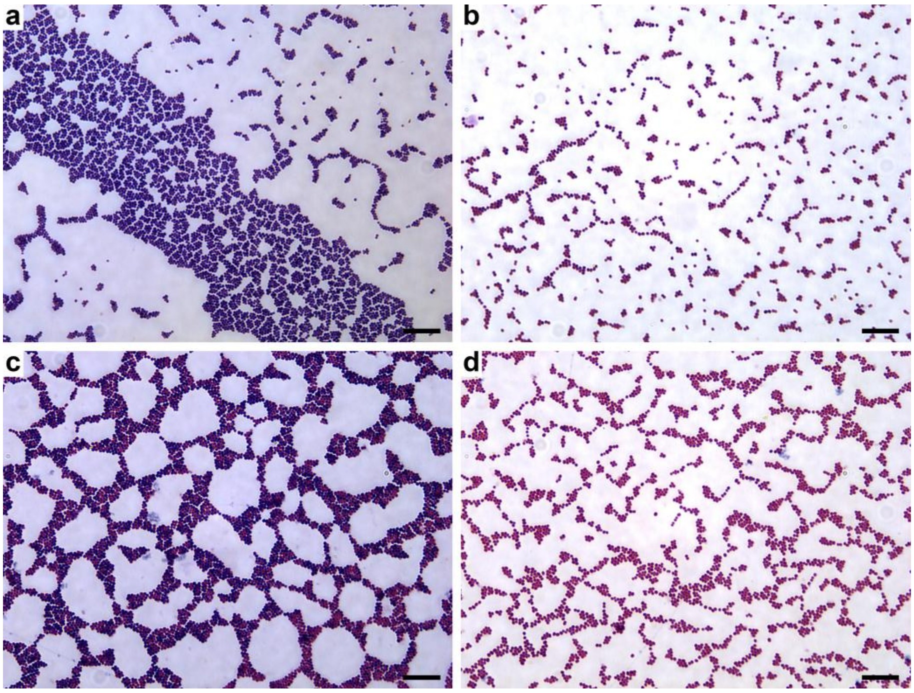

Figure S30 Microscopic images of MSSA strain 21R06006 after Gram staining. (a) Staining results without oxacillin sodium salt. (b-d) Three parallel experiments using oxacillin sodium salt. Scale bars = 10 μm.

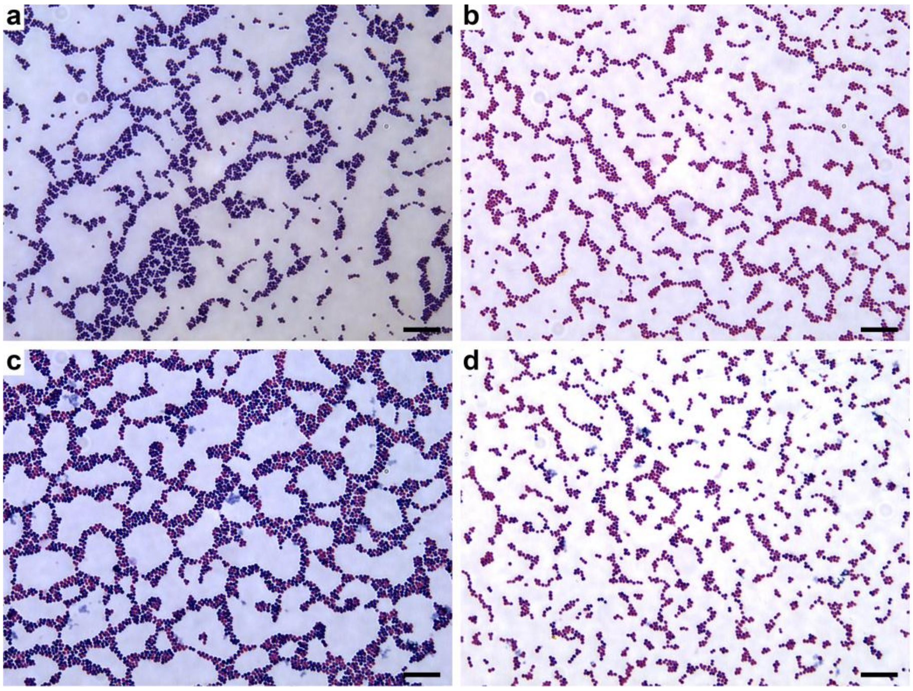

Figure S31 Microscopic images of MSSA strain 21R06100 after Gram staining. (a) Staining results without oxacillin sodium salt. (b-d) Three parallel experiments using oxacillin sodium salt. Scale bars = 10 μm.

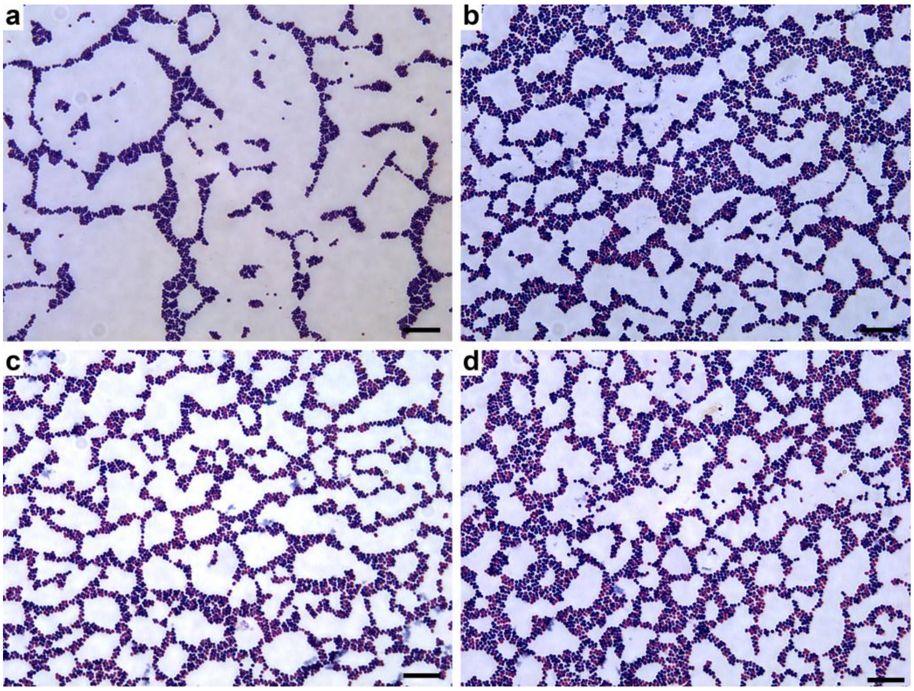

Figure S32 Microscopic images of MRSA strain 21R06320 after Gram staining. (a) Staining results without oxacillin sodium salt. (b-d) Three parallel experiments using oxacillin sodium salt. Scale bars = 10 µm.

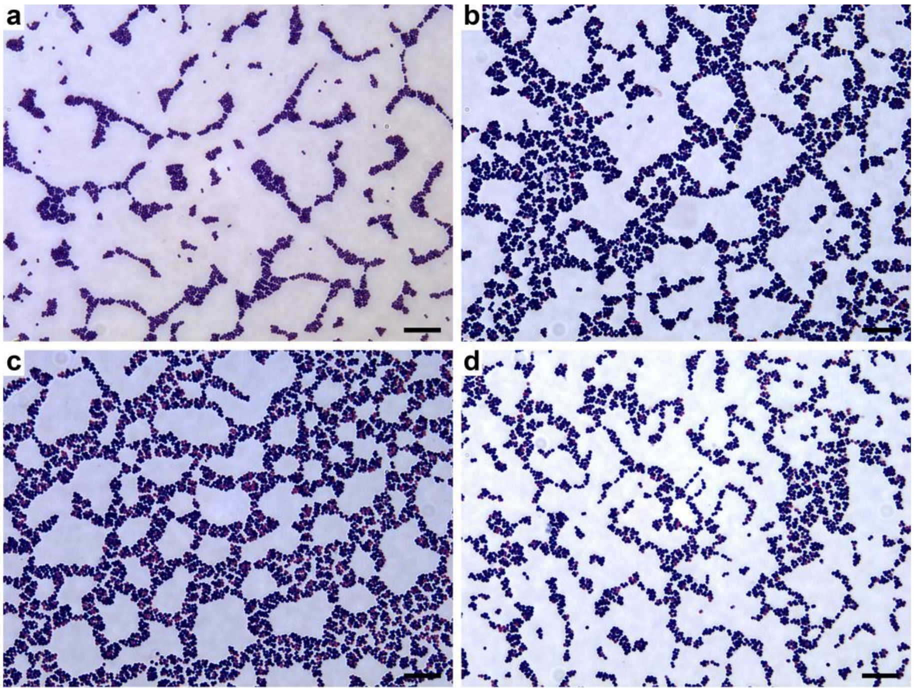

Figure S33 Microscopic images of MRSA strain 21R06322 after Gram staining. (a) Staining results without oxacillin sodium salt. (b-d) Three parallel experiments using oxacillin sodium salt. Scale bars = 10 µm.

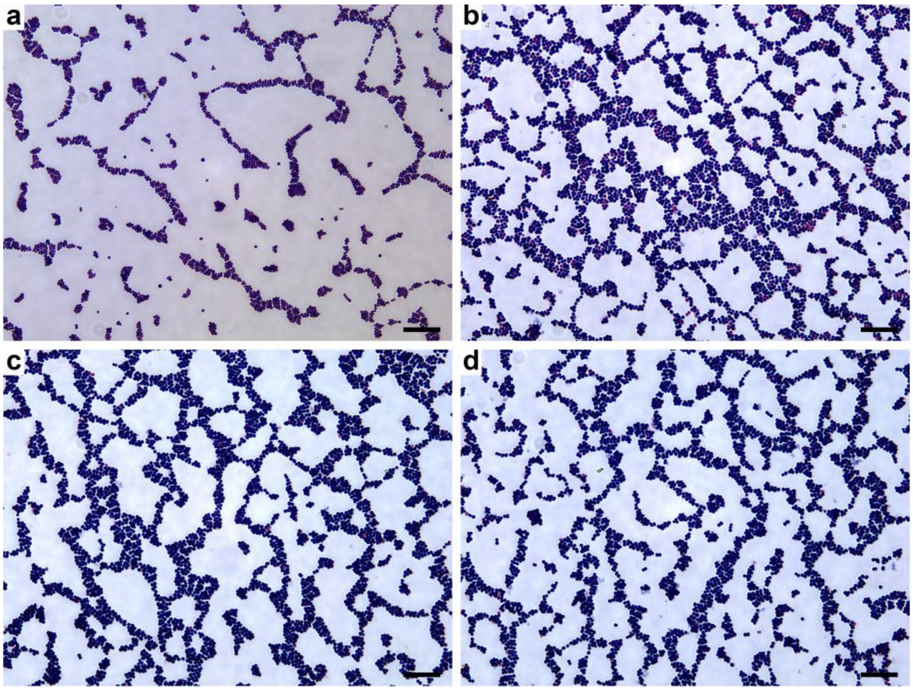

Figure S34 Microscopic images of MRSA strain 21W00424 after Gram staining. (a) Staining results without oxacillin sodium salt. (b-d) Three parallel experiments using oxacillin sodium salt. Scale bars = 10 μm.

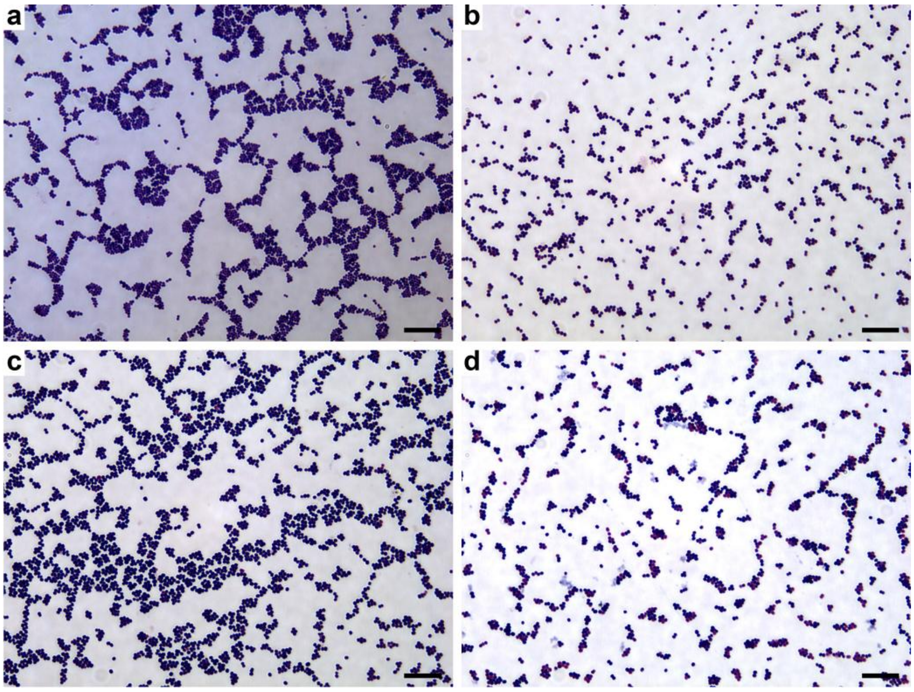

Figure S35 Microscopic images of MRSA strain 21W00469 after Gram staining. (a) Staining results without oxacillin sodium salt. (b-d) Three parallel experiments using oxacillin sodium salt. Scale bars = 10 μm.

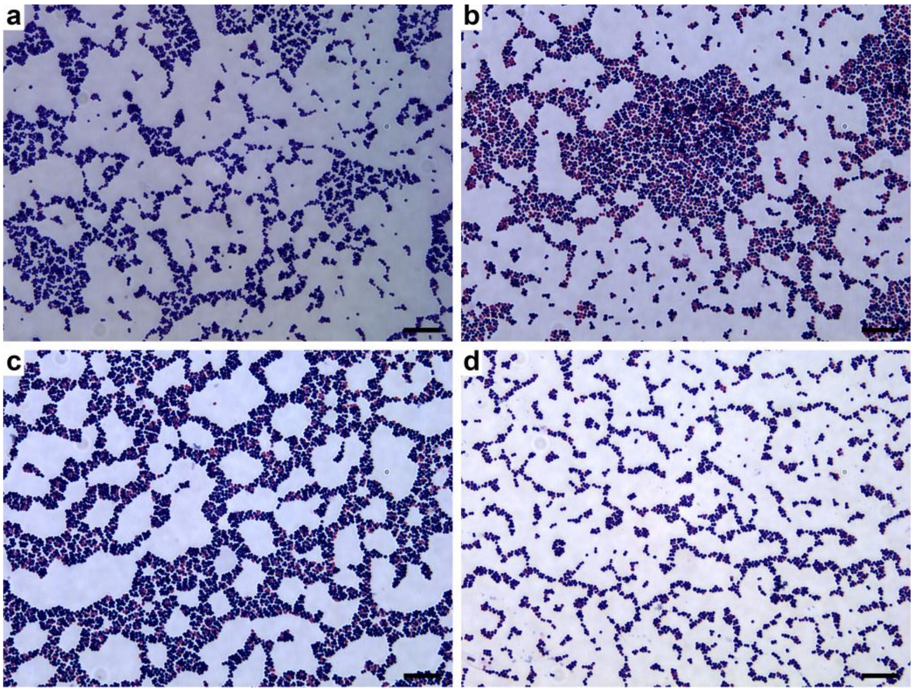

Figure S36 Microscopic images of MRSA strain 21W02896 after Gram staining. (a) Staining results without oxacillin sodium salt. (b-d) Three parallel experiments using oxacillin sodium salt. Scale bars = 10 μm.

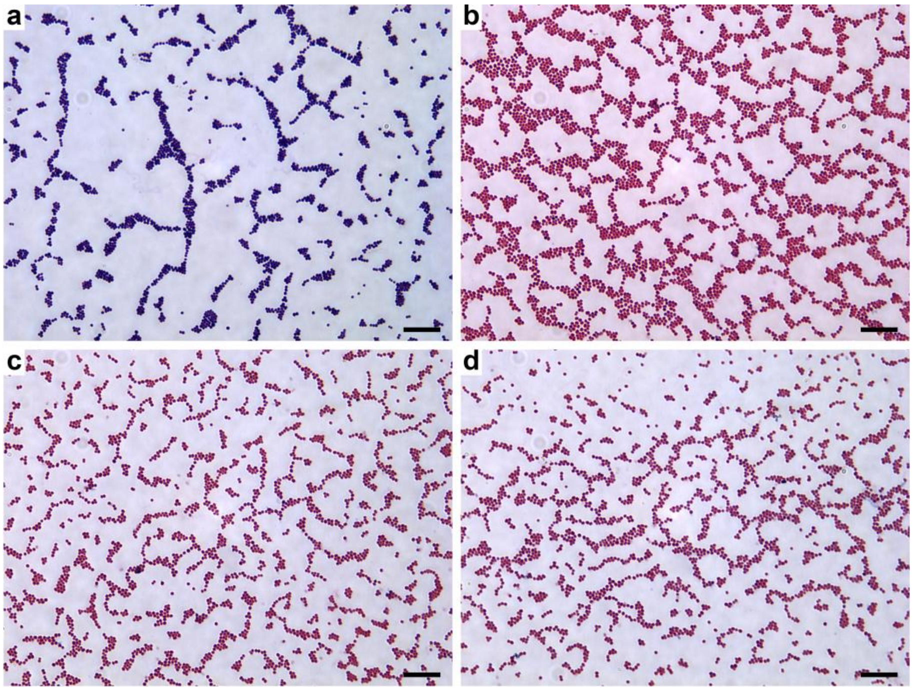

Figure S37 Microscopic images of MSSA strain 21W02902 after Gram staining. (a) Staining results without oxacillin sodium salt. (b-d) Three parallel experiments using oxacillin sodium salt. Scale bars = 10 μm.

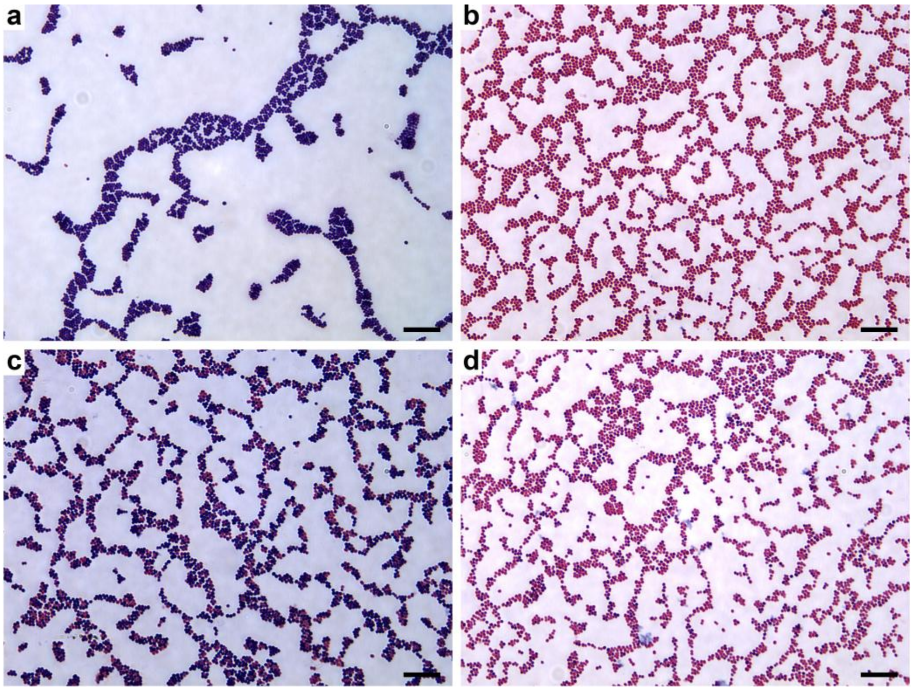

Figure S38 Microscopic images of MSSA strain 21W02928 after Gram staining. (a) Staining results without oxacillin sodium salt. (b-d) Three parallel experiments using oxacillin sodium salt. Scale bars = 10 µm.

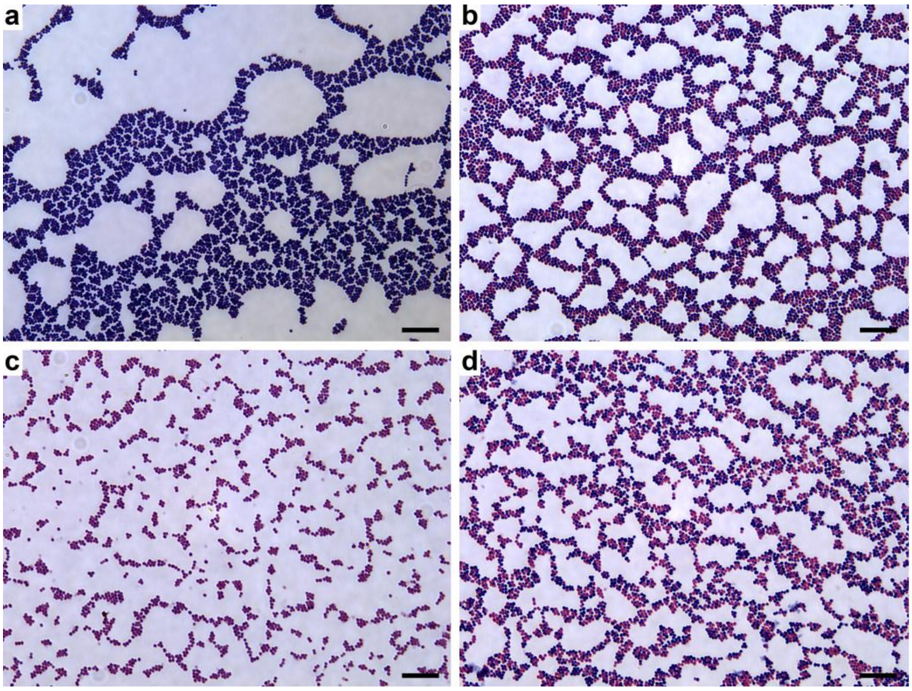

Figure S39 Microscopic images of MSSA strain 21W02970 after Gram staining. (a) Staining results without oxacillin sodium salt. (b-d) Three parallel experiments using oxacillin sodium salt. Scale bars = 10 µm.

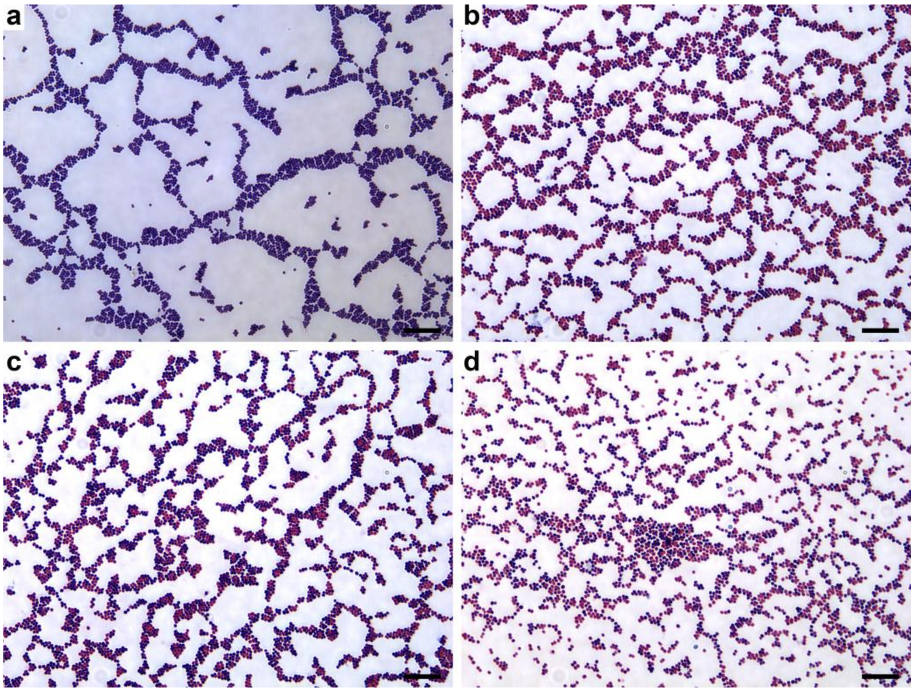

Figure S40 Microscopic images of MSSA strain 21W02977 after Gram staining. (a) Staining results without oxacillin sodium salt. (b-d) Three parallel experiments using oxacillin sodium salt. Scale bars = 10 μm.

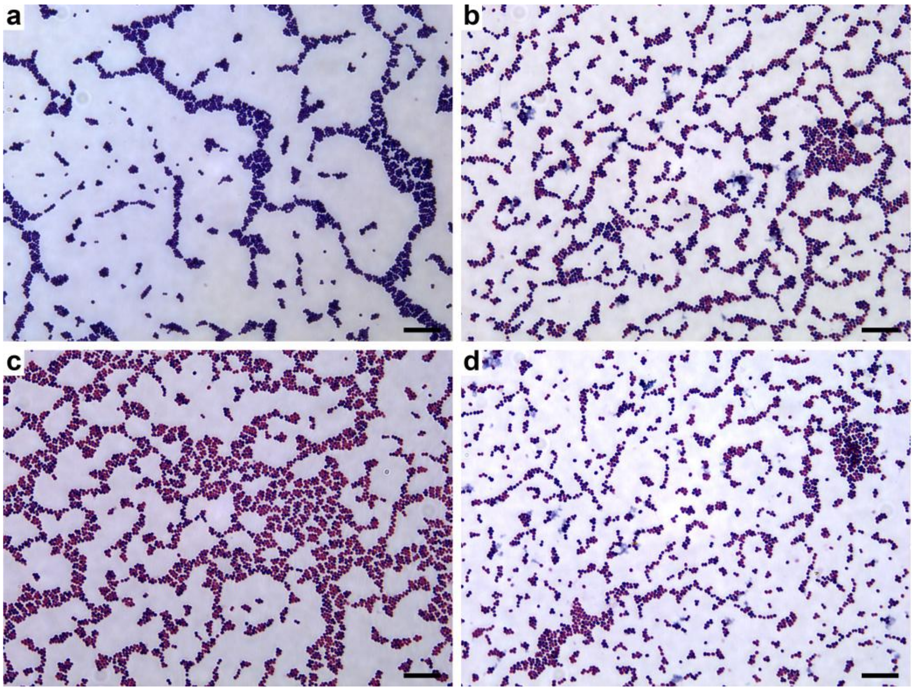

Figure S41 Microscopic images of MSSA strain 21W02987 after Gram staining. (a) Staining results without oxacillin sodium salt. (b-d) Three parallel experiments using oxacillin sodium salt. Scale bars = 10 μm.

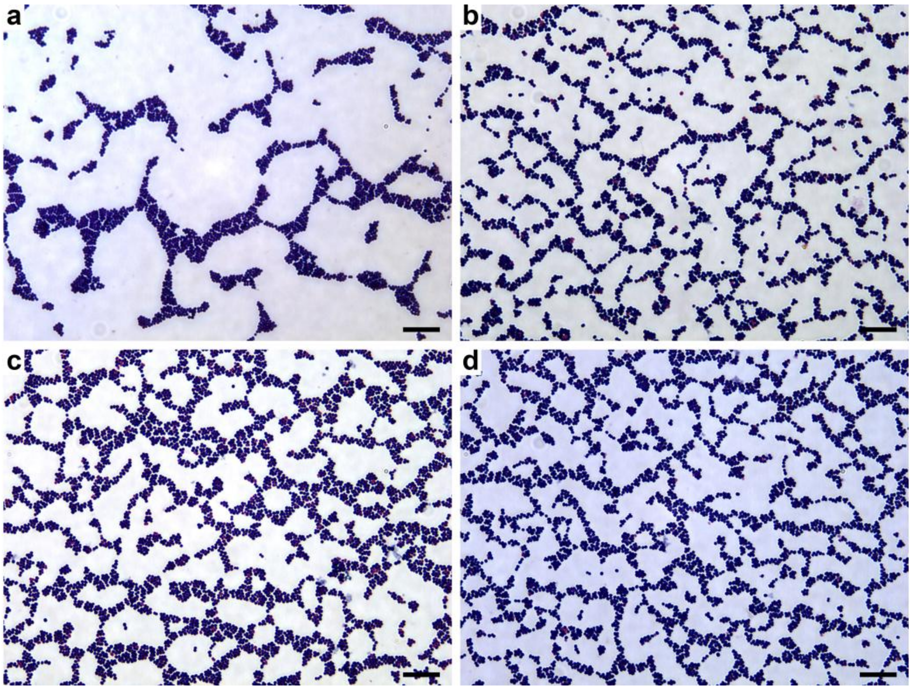

Figure S42 Microscopic images of MSSA strain 21W02998 after Gram staining. (a) Staining results without oxacillin sodium salt. (b-d) Three parallel experiments using oxacillin sodium salt. Scale bars = 10 μm.

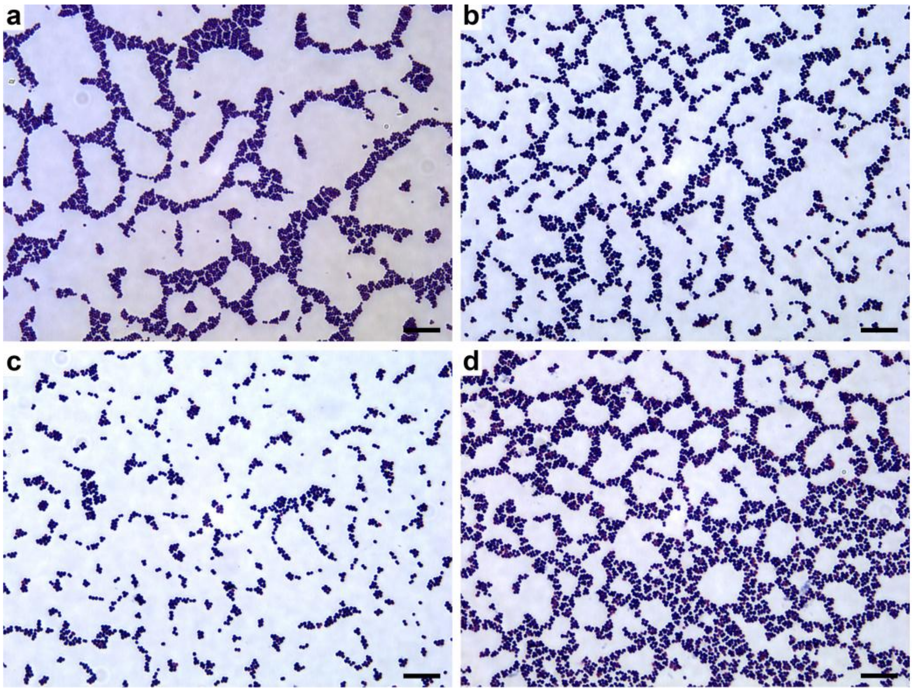

Figure S43 Microscopic images of MRSA strain 21W03011 after Gram staining. (a) Staining results without oxacillin sodium salt. (b-d) Three parallel experiments using oxacillin sodium salt. Scale bars = 10 μm.

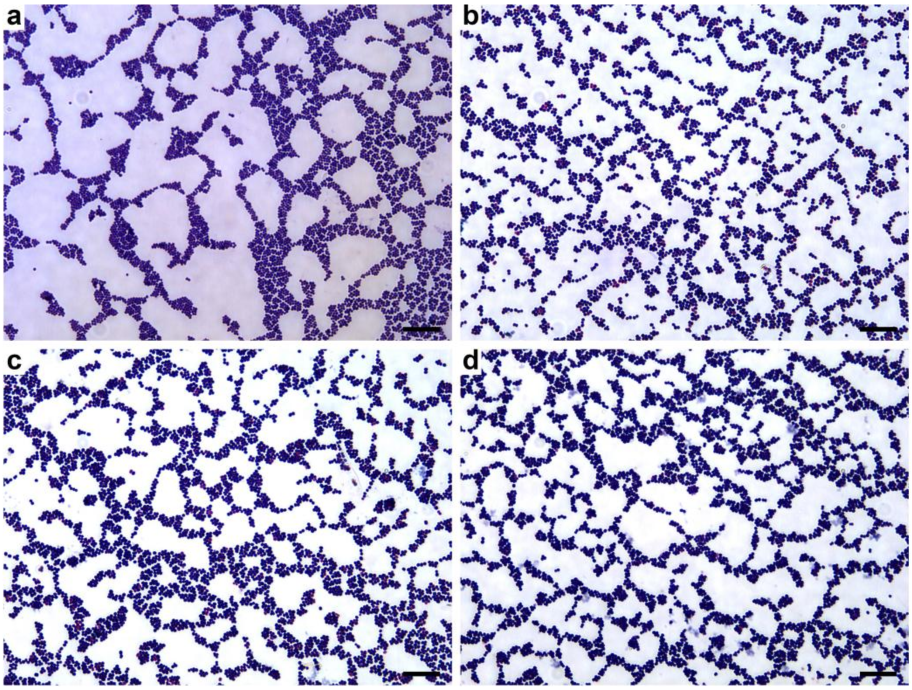

Figure S44 Microscopic images of MRSA strain 21W03078 after Gram staining. (a) Staining results without oxacillin sodium salt. (b-d) Three parallel experiments using oxacillin sodium salt. Scale bars = 10 μm.

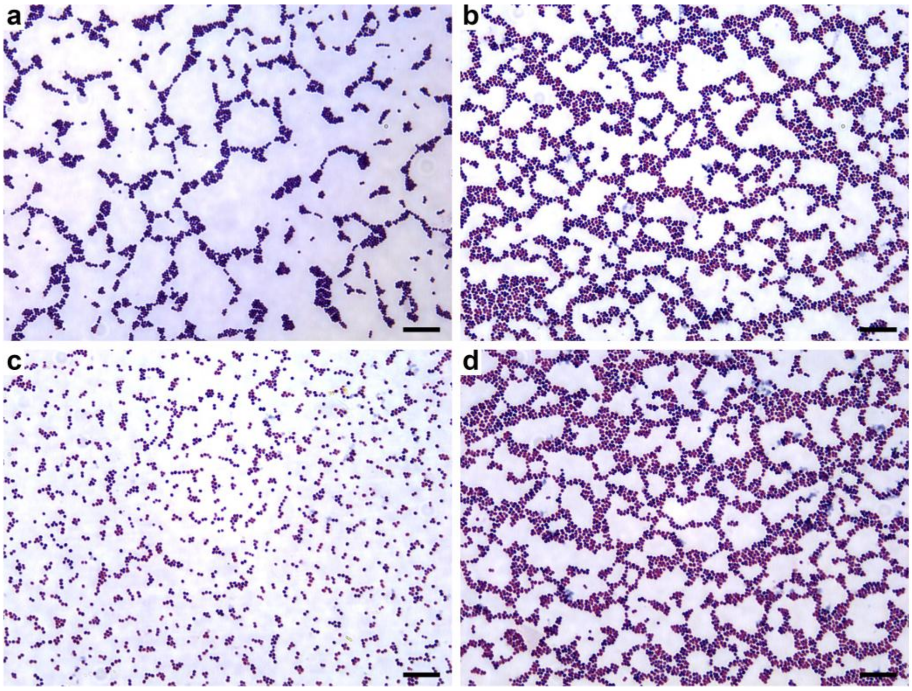

Figure S45 Microscopic images of MSSA strain 21W03097 after Gram staining. (a) Staining results without oxacillin sodium salt. (b-d) Three parallel experiments using oxacillin sodium salt. Scale bars = 10 μm.

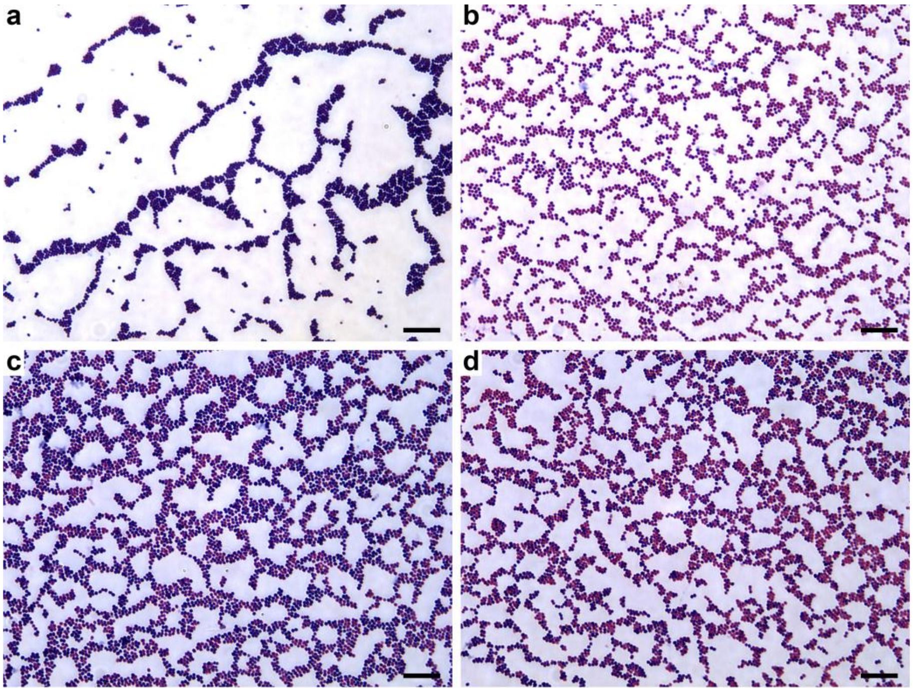

Figure S46 Microscopic images of MSSA strain 21W03127 after Gram staining. (a) Staining results without oxacillin sodium salt. (b-d) Three parallel experiments using oxacillin sodium salt. Scale bars = 10 μm.

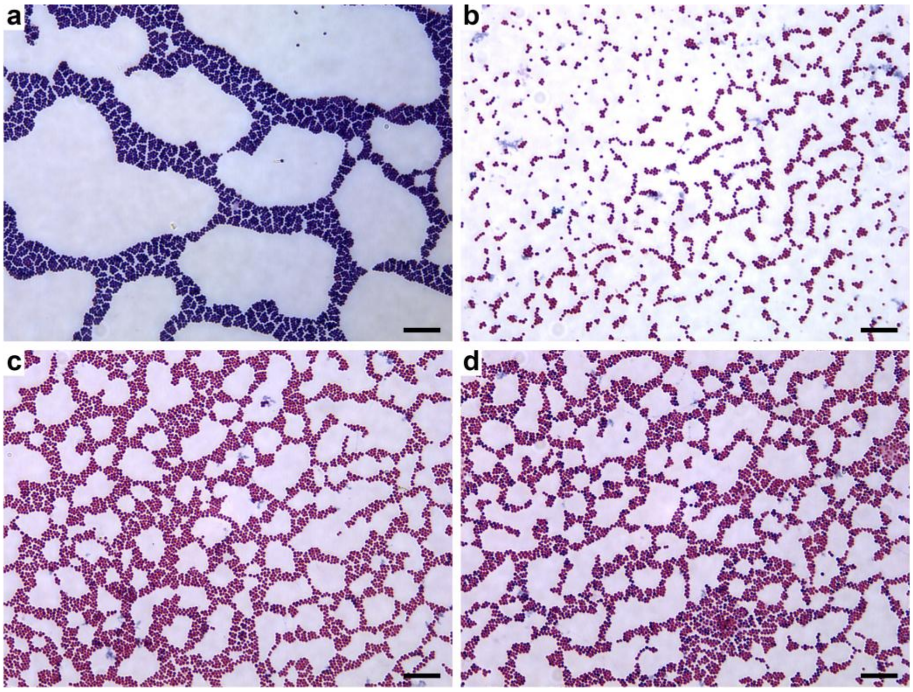

Figure S47 Microscopic images of MSSA strain 21W03210 after Gram staining. (a) Staining results without oxacillin sodium salt. (b-d) Three parallel experiments using oxacillin sodium salt. Scale bars = 10 μm.

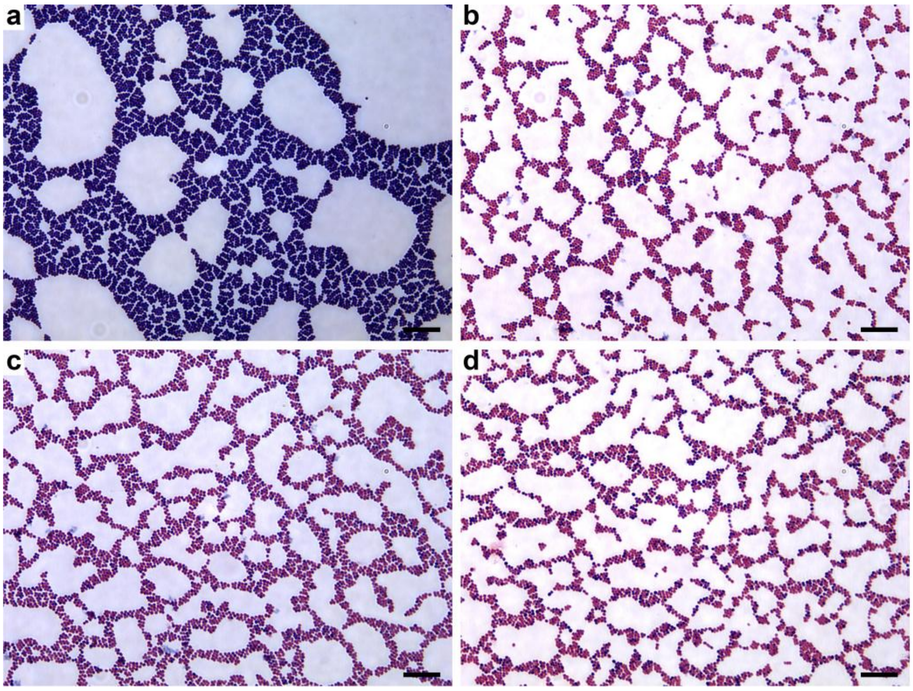

Figure S48 Microscopic images of MSSA strain 21W03229 after Gram staining. (a) Staining results without oxacillin sodium salt. (b-d) Three parallel experiments using oxacillin sodium salt. Scale bars = 10 μm.

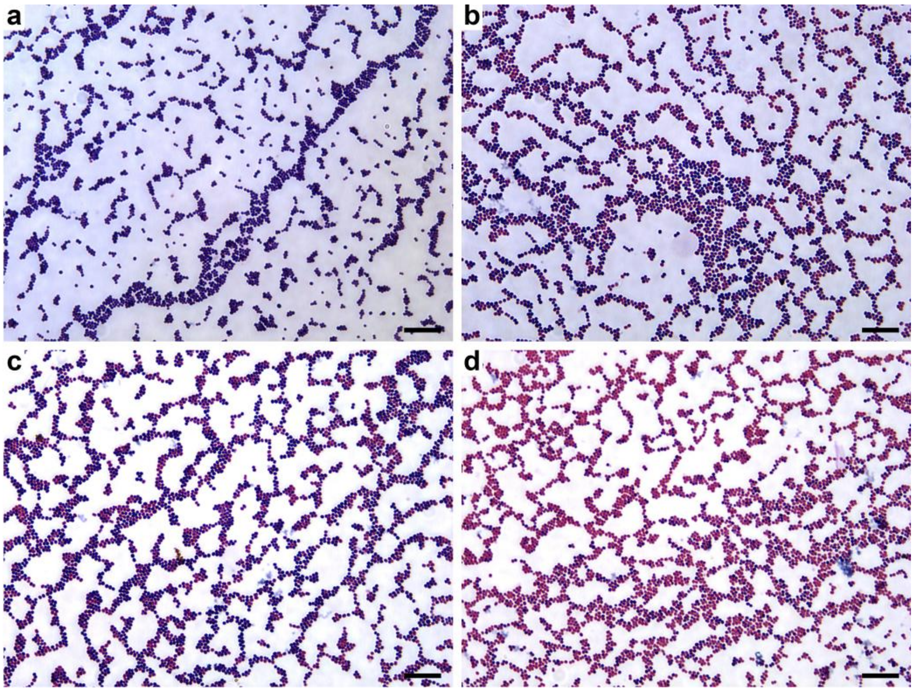

Figure S49 Microscopic images of MSSA strain 21W03248 after Gram staining. (a) Staining results without oxacillin sodium salt. (b-d) Three parallel experiments using oxacillin sodium salt. Scale bars = 10 μm.

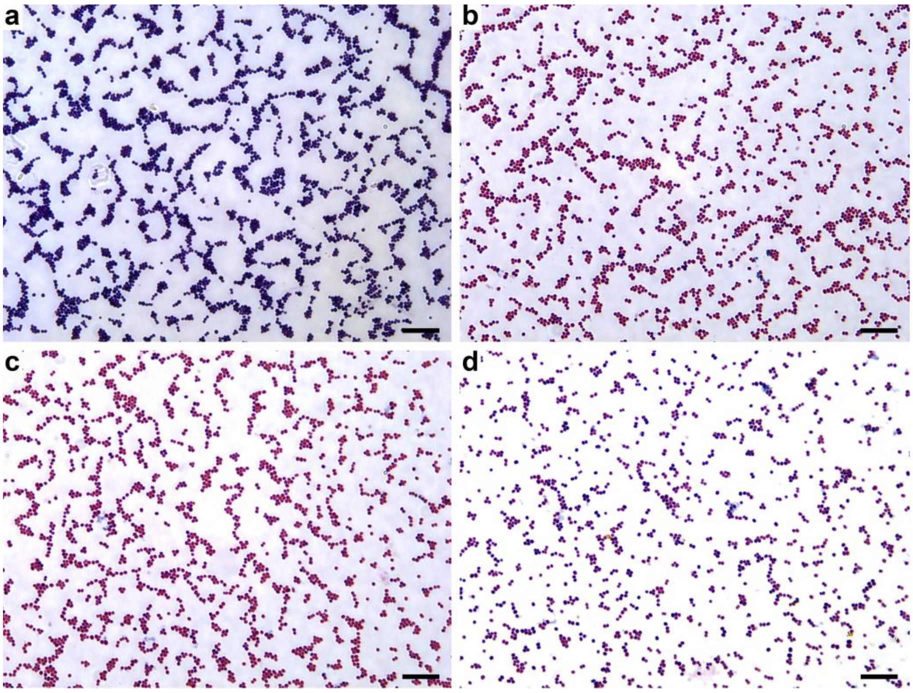

Figure S50 Microscopic images of MSSA strain 21W03255 after Gram staining. (a) Staining results without oxacillin sodium salt. (b-d) Three parallel experiments using oxacillin sodium salt. Scale bars = 10 µm.

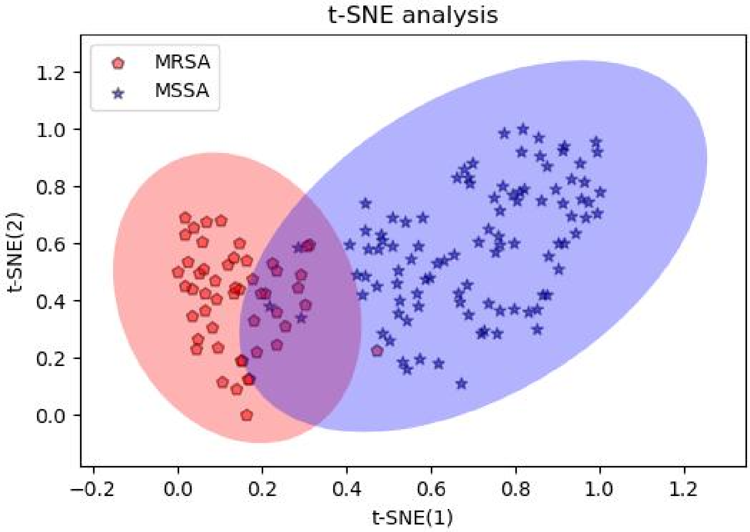

Figure S51 t-SNE analysis of the two classes.

Table. S1 The results of the minimal inhibitory concentration of 50 clinical of *S. aureus*.

Strain number	MRSA	MIC (μg/mL)
21B03641	+	≥4.0
21B06749	+	≥4.0
21B07044	+	≥4.0
21B07569	+	≥4.0
21B08249	+	≥4.0
21B09565	-	≤0.25
21B09625	-	0.5
21B09710	-	0.5
21B09730	-	0.5
21B09791	-	0.5
21B11043	-	≤0.25
21B11183	+	≥4.0
21B11780	-	0.5
21B11864	-	0.5
21C01158	+	≥4.0
21C01161	-	0.5
21C01222	-	0.5
21C01383	-	0.5
21R04907	-	≤0.25
21R04987	-	0.5
21R05171	-	0.5
21R05220	+	≥4.0
21R05221	-	≤0.25
21R05288	-	0.5
21R05322	-	1.0
21R05333	+	≥4.0
21R05342	-	0.5
21R05422	+	≥4.0
21R05646	-	0.5
21R06006	-	0.5
21R06100	-	≤0.25
21R06320	-	0.5
21R06322	+	≥4.0
21W00424	+	≥4.0
21W00469	+	≥4.0
21W02896	+	≥4.0
21W02902	-	0.5
21W02928	-	≤0.25
21W02970	-	0.5
21W02977	-	0.5
21W02987	-	≤0.25

21W02998	+	≥4.0
21W03011	+	≥4.0
21W03078	+	≥4.0
21W03097	-	0.5
21W03127	-	≤0.25
21W03210	-	0.5
21W03229	-	0.5
21W03248	-	≤0.25
21W03255	-	≤0.25

The “+ ” means MRSA, and “-” means MSSA.

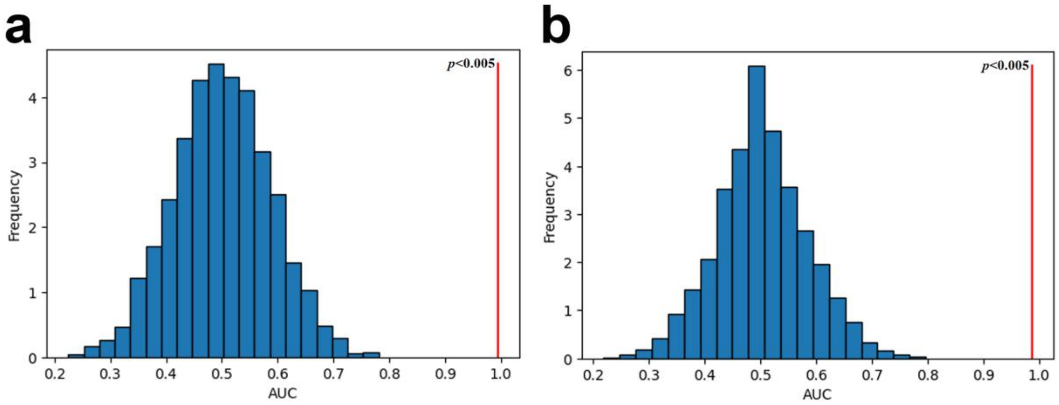

Figure S52 Permutation tests in machine learning. Specifically, the distribution of the AUC was calculated by 5000 random permutations of (a) LDA or (b) ANN. The p-values of these two classifiers were all < 0.005, which means that there was no overfitting.
